# Data quality assessment framework for critical raw materials. The case of cobalt

**DOI:** 10.1016/j.resconrec.2019.104564

**Published:** 2020-06

**Authors:** María Fernanda Godoy León, Jo Dewulf

**Affiliations:** Research Group STEN, Ghent University, Coupure Links 653, 9000, Ghent, Belgium

**Keywords:** Data quality assessment, Critical raw materials, Cobalt, Criticality assessment, Life cycle, Material flow analysis

## Abstract

Critical Raw Materials (CRMs) require a deep understanding of their societal metabolism, for which robust data and information are needed. However, despite the efforts to build reliable data, some CRMs such as cobalt, are still characterised by lack of data harmonization, lack of connection between datasets, and significant data unavailability. Together with data gaps filling, data quality is a crucial aspect to improve Material Flow Analysis (MFA) and Criticality Assessment (CA). Nevertheless, most of the methodologies for Data Quality Assessment (DQA) are not designed for these tools, but for others, e.g. life cycle assessment. The current research addresses the following challenges; a better understanding of the societal metabolism of CRMs; the development and implementation of DQA in MFA and CA; and a better understanding of the available data related to current cobalt flows in the EU technosphere. The underlying life cycle phases of CRMs within the technosphere were identified, together with 15 key parameters. A new DQA matrix was developed, which was subsequently applied to the full dataset collected for cobalt. The dataset was built considering seven high-end applications of cobalt. More than 300 values were gathered, which were analysed in function of different aspects, such as the country/region, and year. Through the data analysis and the application of the DQA framework, data gaps were identified due to low availability and/or low quality. It was concluded that the main deficiency of cobalt data is its reliability, due to lack of information regarding its generation method, and the incomplete stakeholder coverage.

## Graphical abstract

**Figure:**
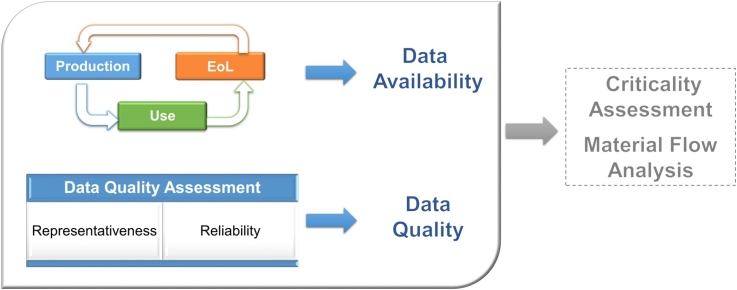

## 1 Introduction

The growing population and the dynamic demand for raw materials of the last decades have raised the global material extraction, leading to a growing pressure on natural resources. This has caused not only harm to the natural environment and human health, but also a growing concern about material supply security. In this line, sustainable resource supply and management have become increasingly important in the last years, standing out as top priorities on the international political agenda (UNEP, 2016). To address these issues, several initiatives have been launched in different parts of the world, e.g. the International Research Panel (IRP) of the United Nations, the Raw Materials Initiative of the European Union (EU) (European Commission, 2018d), the Critical Materials Strategy of the USA (USGS, 2018), the Critical Minerals Strategy of Australia (Commonwealth of Australia, 2019), and the Resource Securement Strategies of Japan (Hatayama and Tahara, 2014). These initiatives seek to build knowledge about the use of natural resources, addressing different aspects such as supply and demand, resource efficiency, and recycling. The latter is particularly important, as a way to decrease the demand of virgin material through the production of secondary resources, which also promotes security and decreases dependence on trade (IRP, 2017).

As part of their strategies, the EU, the USA, Australia, and Japan have identified a number of critical/strategic materials, characterizing them by economic importance and supply risk. In 2017, the EU developed its third assessment of Critical Raw Materials (CRMs), implementing a revised methodology and covering substantially more materials than in the previous assessments (78 in 2017, compared to 41 in 2011, and 54 in 2014) (European Commission, 2017a). Together with the identification of these materials, a deep understanding of their societal metabolism is required, considering not only their flows but also the parameters and data needed for the estimation of the flows, which creates the need for robust information and data. Consequently, several programs, projects, and studies have been developed to address this need. The EU has established or supported different data initiatives to either expand or improve data routinely collected by DG-Eurostat, or through the DG-JRC’s Raw Materials Information System (RMIS), the DG Growth’s Material System Analysis (MSA), and a number of EU-funded projects, e.g. PROSUM, MICA, ORAMA, SCRREEN. Despite these efforts, there is still lack of data harmonization, lack of connection between datasets, and a significant unavailability of data (data gaps) for specific materials (RPA, 2012; Huisman et al., 2017).

Another aspect that needs further development in Criticality Assessment (CA) and Material Flow Analysis (MFA) is the evaluation of the quality of the data used in these studies. On the one hand, assessing and stating the quality of the data used in these (and any other) studies contributes to a better documentation of the data, which according to Pauliuk et al. (2015) and Hertwich et al. (2018) provides more transparent publications and a higher accessibility and reusability of the data. On the other hand, the results of the studies are directly affected by data quality. Thus, studies developed with data gaps and low data quality will lead to results characterised by low robustness and reliability.

Some characteristics, such as temporal and geographical representativeness of the data, are key to assess their quality. In the last decades, some methodologies have been developed for Data Quality Assessment (DQA) applied in Life Cycle Assessment (LCA) and, in a less extent, in MFA. In 1996, Weidema and Wesnæs developed one of the most known methods to evaluate data quality in LCA, the so-called Pedigree-matrix (Weidema and Wesnaes, 1996). This method gives a semi-quantitative indication of reliability; completeness; and temporal, geographical and further technological correlations, through five Data Quality Indicators (DQIs). It has been widely used in LCA, and it has served as basis for the development of similar methodologies elsewhere. Recently, the USA and the EU also established their own matrix developed for LCA, using similar indicators to those proposed in 1996 (Manfredi et al., 2012; Edelen and Ingwersen, 2016). Regarding MFA, different studies have been published addressing data uncertainty (Laner et al., 2014, 2015a; 2015b; Džubur et al., 2017; Allesch and Rechberger, 2018). However, to the best of the authors’ knowledge, semi-quantitative DQA through quality indicators has only been developed by Laner et al. (2015a), who proposed a similar method to that of the Pedigree-matrix; and by BIO by Deloitte, who used a semi-quantitative method in their Material System Analysis (MSA) study (BIO by Deloitte, 2015) to assess the reliability of the data, but not its representativeness. The European Commission also counts with a simple approach to assess que quality of the data used in their studies of CA (European Commission, 2017a).

A specific case of CRM is cobalt (Co), a critical/strategic material for the EU, the USA, Australia, and Japan. This metallic element is used in alloys (e.g. permanent magnets, hard metals, superalloys, electrodeposited alloys in metal coatings) and in the production of chemicals (e.g. pigments, catalysts, paint driers, trace metal additives for agricultural and medical use, rechargeable batteries) (Donaldson and Beyersmann, 2012). The main reasons for interest and concern about Co are its use in rechargeable batteries, key element in the transition from fossil fuels to sustainable energy sources; and that it is mainly mined in the Democratic Republic of Congo, a country considered politically unstable (World Bank, 2019).

Due to its criticality, several reports have been commissioned to fully or partially address the supply, demand, stock and flows of Co (National Research Council, 1983; OTA, 1983; Shedd, 1993; RPA, 2012; BIO by Deloitte, 2015; Alves Dias et al., 2018; European Commission, 2018b). One example is the Study on Data Needs for a Full Raw Materials Flow Analysis, commissioned by the EU and published in 2012, which was developed for 21 raw materials. The objective of this study was to identify the information and data needs for a complete analysis of the raw materials flow at the European level, assessing available data related to material flows, data gaps and bottlenecks (RPA, 2012). However, this study was not exhaustive, due to time and budget constraints. Specifically for Co, most of the available data was related to primary resources, but little was found regarding waste and secondary resources. A follow-up of this study was the MSA report, published in 2015. This study consisted in a map of the flows through the economy, including the inputs and movements within it, additions to stock, and end-of-life through either disposal or recovery (BIO by Deloitte, 2015). To the best of the authors’ knowledge, these reports are the only two sources of compiled data related to Co flows in Europe that consider different applications of the metal. Other studies focus generally on the use of Co in Li-ion batteries, and most of the other applications are poorly described in terms of the metal flows. Nevertheless, their main findings and results are in terms of stocks and flows, and little information is given regarding the parameters required to calculate these values. Crucial parameters such as production yields, collection rates, and recycling efficiencies are not systematically provided. Another limitation of these studies is their reliance on data from other countries (mainly the USA), and from rather outdated reports. Moreover, in the MSA report, the value of some key parameters such as the percentages of waste hoarded by consumers and the hoarding periods were unknown, requiring several assumptions and hypotheses. Both reports provide reliable data and results related to the extraction and processing of primary resources; but not for the steps of use, collection and recycling, where data gaps and results with low robustness are indicated.

This research aims to address three different challenges. First, there is a need for a better understanding of the societal metabolism of materials, in particular of CRMs. Second, it is fundamental to further develop and implement DQA in the application of CA, MFA, and similar studies, assessing the reliability and representativeness of the data. Third, there is a need for a better understanding of data related to Co flows in the technosphere, especially regarding secondary resources. Within this context, the goals of the research are (i) to develop a flowchart applicable to CRMs, identifying key parameters for the estimation of their flows; (ii) to develop a new DQA framework; and (iii) to apply the developed schemes to explore and assess data regarding Co.

## 2 Methodology

### 2.1 Flowchart of CRMs

To date, there are general flowcharts illustrating the flows of services and/or goods (e.g. MMSD Project, 2002; Dewulf et al., 2015; Ellen MacArthur Foundation, 2015; Rogers et al., 2015). These flowcharts follow the classic structure ‘extraction/refining - manufacturing - use - end-of-life’ without considering key phases such as hoarding (hibernation). Moreover, they do not relate the phases with the parameters required to estimate material flows. For CRMs, available flowcharts (BIO by Deloitte, 2015; European Commission, 2017b) are rather simple, without addressing in detail all the involved life cycle phases of the materials. In 2014, Nakamura et al. (2014) presented an advanced flowchart for materials in general, considering not only material and processes, but also some parameters required to estimate the flows. In the present research, a detailed and refined flowchart was developed focusing on secondary resources (primary resources are out of the scope), indicating phases and sub-phases of the life cycle of CRMs, together with the parameters required to estimate and assess their flows. The flowchart was developed based on the mentioned studies related to CA and/or MFA (Nakamura et al., 2014; BIO by Deloitte, 2015; European Commission, 2017b), adding new phases and parameters according to the literature review.

### 2.2 Development of a new DQA framework

Data quality has been defined as “characteristics of data that relate to their ability to satisfy stated requirements” (ISO, 2006). These requirements are established through Data Quality Goals (DQGs), which describe the ideal representativeness and completeness of the data according to the boundaries of the study. DQGs can be related to the year of measurement, the geographical area, the data completeness, and the technology being modelled. DQA is the evaluation of the collected data compared to the defined DQGs (Weidema and Wesnaes, 1996; Edelen and Ingwersen, 2016).

Within this context, a new framework for DQA was built. A semi-quantitative method was established starting from the schemes proposed by Weidema and Wesnæs (1996), Manfredi et al. (2012); Laner et al. (2015a), and Edelen and Ingwersen (2016). A comparative table of these schemes is provided in SI.

The new framework was built by combining different characteristics of the aforementioned methods. Two main aspects of data were identified, representativeness (depending on the DQGs of the case study) and reliability (independent of the DQGs, inherent of the data itself). The data representativeness was defined in function of the temporal, geographical and technological coverage. Temporal and geographical coverage assess the country or region and the year for which the datum is representative, respectively. The technological coverage refers to the congruence of the available data with respect to the evaluated product (values related to the exact same product are evaluated with a higher quality compared to those for products that strongly deviate from the concerned application).

To assess reliability, two options (A and B) were defined depending on the type of datum to assess. Option A is applicable to measurements and calculations, and two features were evaluated: the method through which the datum was generated, and the validity of the value. Data can be generated through measurements, calculations or estimates. Measurements are considered more reliable than calculations, and calculations based on measurements are more reliable than calculations based on assumptions. The validity refers to how well the value is accepted or acknowledged, and it was evaluated based on the range of involved stakeholders. The interested stakeholders were defined as companies, industry associations, governmental institutions, universities, research institutes or centres, and individual experts. Option B was established for estimates, which are generally used in case published data or measurements are unavailable. Its validity depends on the transparency of the generation process and the knowledge of the expert about the subject in question. Estimates correspond to values obtained through personal communication or indicated as an assumption.

Based on these different aspects of data quality, six DQIs were established, scoring from 1 (highest quality) to 4 (lowest quality): Geographical correlation, Temporal correlation, and Technological correlation (to assess representativeness), and Generation method, Validity, and Expert estimate (to assess reliability). The framework is presented and discussed in Section 3.2.

### 2.3 Case study case on cobalt

Seven high-end applications of Co were identified in this study: batteries, catalysts, intentionally dissipative uses (e.g. pigments), hard metals, magnets, superalloys, and other metallic uses (e.g. tool steels and semiconductors). The application batteries was divided in three sub-applications: portable batteries, mobility batteries, and unspecified Co batteries. The main focus was Li-ion batteries. In the case of catalysts, four sub-applications were studied: for hydroprocessing, for hydroformylation, for the production of polyester (PET) precursors, and unspecified Co catalysts. In SI a brief description of each application is given.

Even though the purpose was to assess two sub-applications of batteries (portable and mobility) and three of catalysts (for hydroprocessing, for hydroformylation, and for the production of PET precursors), the categories unspecified Co batteries and unspecified Co catalysts were needed in the analysis. These categories were established since some of the information was not specific for a type of battery or a type of catalyst. Moreover, in the case of batteries some values were applicable to both types.

### 2.4 Data exploration, collection and validation

The availability of Co data was explored exhaustively, evaluating them thoroughly in function of their representativeness for Europe at present. The research focused on physical Co flows, concerning the metal embedded in final products. The scope of the study was data related to physical aspects of the flows considering the parameters defined in Section 3.1; with a special focus on end-of-life products, and secondary resources (primary resources and economic inputs/outputs are out of scope).

Datasets about Co were built implementing the following three steps:

• Exploration: An exhaustive literature research was carried out using web-based searching tools, consulting journals, reports, books, conference and course presentations, proceedings, websites, and patents. The data exploration was done for Co and for each single application. Only sources in English were consulted, published between 1980 and middle 2018.
• Collection: Data and information were selected to be part of the datasets, sorted by application. The focus was to collect data representative for Europe at present time. However, global data and data related to other countries or regions were also considered when found. The time of the data covered the decade of the 70s to the present time. Data were collected from literature and through personal contacts.
• Validation: Companies, industry associations, researchers, and experts were consulted in order to validate the gathered data. The consultation was done through emails, teleconferences, phone meetings and face-to-face meetings. Key contributors were the Cobalt Institute (CI), the Committee of PET Manufacturers in Europe (CPME), the European Battery Recycling Association (EBRA), and the UK Magnetics Society. In SI all consulted institutions (for collection and validation) are listed per application.

**Fig. 1:**
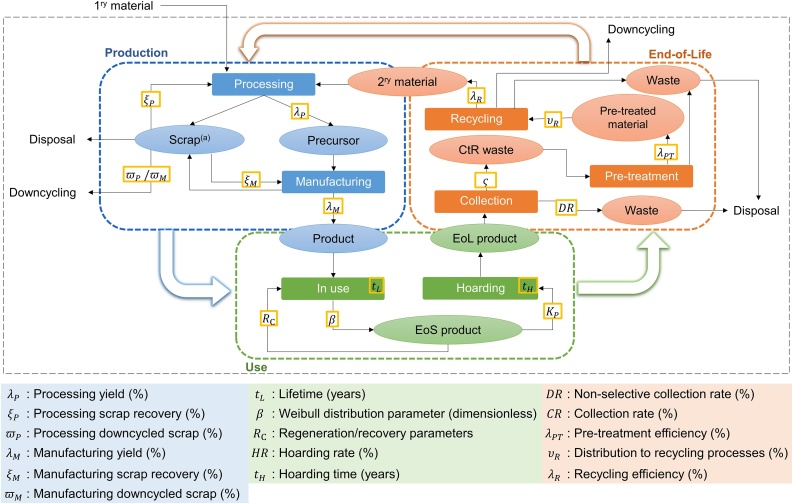
Identified life cycle phases (colour dashed rectangles) and parameters (yellow rectangles) for each one. Ovals represent materials, products or waste; solid rectangles represent processes or sub-phases of the life cycle, black dashed rectangle represents the scope (primary raw material is out of scope). The percentages are weight percentages. EoL: End-of-Life, EoS: End-of-Service, CtR: Collection-to-Recycling. (a) New and prompt scrap.

Each collected value was characterized according to the covered geographical area, the year for which the datum is representative, the method to generate the datum, the communication format, and the life cycle phase (according to Fig. 1). This was done based on the information of the original source.

## Geographical category

Six groups were established: Europe/EU, USA, Japan, Global, Other countries, and Not available. Europe/EU covers data regarding Europe as continent, the EU as a whole (EU28), and subdivisions of the EU (e.g. EU25, EU5, and single member countries). It also considers data representative for the EU28 plus Switzerland and Norway. Global considers data representative for the whole world, and Other countries includes data from Brazil, India, South Africa, and Turkey. Not available consists of the data for which the source does not report or does not clarify the country or region that it covers.

## Temporal category

Data were grouped in Before 2004, 2004–2008, 2009–2013, and 2014-2018. Not available comprises the data for which the source does not report or does not clarify the year that it covers. In some sources, the data were indicated for a period (e.g. 2006–2012). In this case, the latest year was considered in the classification.

## Generation method category

Six groups were established: Experimental/Survey, Assumption/Estimation, Modelled, National statistics, Reported by company, and Not available. Experimental/Survey includes data produced through experiments at laboratory or pilot scale, and through surveys applied to consumers or companies. Assumption/Estimation comprises data indicated as an assumption or estimated by an expert. Modelled refers to data generated through mathematical models or calculated based on raw data. National statistics consists of data generated by countries, reported to (inter)national statistical offices. Reported by company refers to data informed by specific companies. Not available consists of the data for which the source does not report or does not clarify how the datum was produced.

National statistics and data reported by companies can be based on data produced through models, experiments and/or estimations, but normally this information is not given. For this reason, and because these data possess a certain reliability, they were assessed in a different category.

## Communication format category

Five groups were established: Peer-reviewed, Report, Personal communication, Conference or course, and Other. Peer-reviewed includes data published in peer-reviewed scientific journals. Reports comprises data published in reports, developed by private or public institutions. Personal communication refers to data given through direct contact with an expert. Conference or course refers to data obtained from presentations, proceedings, course notes, or from any material produced during these activities. Other includes data from books, websites, patents, and other non-peer-reviewed sources.

### 2.5 Application of the DQA framework on the collected dataset

The quality of the collected dataset was assessed by applying the developed DQA matrix. To do so, it was required to define the DQGs of the case study, in order to assess the representativeness (geographical, temporal and technological coverage) of the data.

## Geographical data quality goal

The goal is to evaluate how representative the data are for the EU. Countries and regions were categorized according to their GNI (Gross National Income) per capita in 2017, in order to assess how comparable they are to the EU from an economic perspective. This approach was used as a way to compare standards of living, which are related to productivity and consumption of a population (The Balance, 2018a, b).

Three thresholds were considered: High income (more than US$12 235 per capita), Upper-middle income (between US$12 235 - US$3 956 per capita), and Lower-middle and low income (less than US$3 955 per capita) (World Bank, 2018). The EU is classified as High income; therefore, data from countries in this category are considered more representative for the EU than data from countries in lower categories.

## Temporal data quality goal

The goal is to obtain data representative of the last 5 years (2014–2018).

## Technological data quality goal

The goal is to obtain data for the applications indicated in Section 2.3. To sort the data, four groups of ‘items’ were established. Group 1 represents the highest quality and group 4 the lowest quality. Following, the categories are described and explained through an example:

• Group 1: Same product (e.g. portable Li-ion battery).
• Group 2: Devices containing the product (e.g. laptop instead of portable Li-ion battery).
• Group 3: Subcategory of the product (or devices containing them) (e.g. portable Co battery instead of portable Li-ion battery).
• Group 4: Mix of related devices (e.g. screen devices, including laptops).

### 2.6 Selection of data with the highest quality

The application of the DQA framework allowed the calculation of the Data Quality Rating (DQR) of each value, which were compared per parameter. Depending on the quality of the available data, the quality level of each parameter was established. To do so, the values showing the highest quality (according to the criteria defined in Table 2) were selected. These values constitute the final dataset showed in Table 3.

**Table 1:** Definition of data quality indicators and qualitative evaluation criteria for the application of score 1–4.

Aspects		Indicator		Definition	Score: 1	Score: 2	Score: 3	Score: 4
**Reliability**	Option A		Generation method	Focus on the methodology used for the datum generation	Value from a direct measurement	Value calculated based on measurements	Value calculated based on assumptions	Methodology of datum generation unknown, no documentation available
			Validity	Acceptance of the datum based on the variety of involved stakeholders in its generation or confirmation	Value obtained from or confirmed by 4 or more types of stakeholders	Value obtained from or confirmed by 3 types of stakeholders	Value obtained from or confirmed by 2 types of stakeholders	Value obtained from 1 type of stakeholder
	Option B		Expert estimate	Reliability of a datum based on the transparency of the generation process and the knowledge of the expert	Formal expert elicitation with (empirical) database – transparent procedure and fully informed experts on the subject	Structured expert estimate with some empirical data available or using transparent procedure with informed experts	Expert estimates with limited documentation and without empirical data available	Educated guess based on speculative or unverifiable assumptions
**Representativeness**	Temporal correlation			Congruence of the available datum and the ideal datum with respect to time reference	Value deviates 5 years or less	Value deviates 6–10 years	Value deviates 11–15 years	Value deviates more than 15 years
	Geographical correlation			Congruence of the available datum and the ideal datum with respect to geographical reference	Value relates to the studied region	Value relates to similar socio-economical region (e.g. High income)	Value relates to moderately different socio-economical region (e.g. Upper-middle income)	Value relates to very different socio-economical region (e.g. Lower-middle income and Low income)
	Technological correlation			Congruence of the available datum and the ideal datum with respect to technology, product, etc.	Value relates to the same product, the same technology, etc.	Value relates to similar technology, product, etc.	Value deviates from technology/product of interest, but rough correlations can be established based on experience or data	Value deviates strongly from technology/product of interest, with correlations being vague and speculative

**Table 2:** Overall data quality level according to the achieved Data Quality Rating (DQR). Same criteria apply for reliability (RL) and representativeness (RP).

DQR/R_L_/R_P_	Data quality level
1.0–1.6	Very high quality
1.7–2.4	High quality
2.5–3.2	Low quality
3.3–4.0	Very low quality

**Table 3:** Data quality results for fifteen parameters related to the life cycle of twelve Co (sub)applications. Representativeness assessed for the EU covering the last 5 years.

## 3 Results and discussion

### 3.1 Flowchart and parameters

The life cycle of CRMs was analysed, establishing a flowchart including the main phases: Production, Use, and End-of-Life (EoL). Each phase was divided in sub-phases. Production considers Processing and Manufacturing, which refer to the processing of the precursor and to the manufacturing of the final product, respectively. Use is divided into In use (phase that considers the useful life period of the product), and Hoarding (phase related to the period between the end of the useful life and the collection of the product, also called hibernation). Finally, End-of-Life is split into three sub-phases: Collection, Pre-treatment, and Recycling.

To assess the flows of these materials in the technosphere, a number of key parameters were identified for each sub-phase. These parameters are listed in Fig. 1, including the yield of the production processes, the lifetime of the products, the hoarding rate of the End-of-Service (EoS) products (dead storage of a product that has reached the end of its use), the collection rate (EoL products collected for recycling), and the distribution of the waste to recycling processes. Further definition of the parameters is available in SI.

### 3.2 New DQA framework

In this section, the modified DQA matrix is presented and discussed. Table 1 shows the developed framework, listing the six indicators together with their definition and scoring criteria.

The overall data quality is calculated as the average score of the applied DQIs, using Eq. 1 (if the reliability of the datum is assessed through Option A) or Eq. 2 (if the reliability of the datum is assessed through Option B).

(1) $$DQR=\frac{Gm+V+T+G+Tch}{5}$$

(2) $$DQR=\frac{E+T+G+Tch}{4}$$

Where DQR is Data Quality Rating, Gm is Generation method, V is Validity, T is Temporal correlation, G is Geographical correlation, Tch is Technological correlation, and E is Expert estimate.

Similarly, the reliability ($R_{L}$) and representativeness ($R_{P}$) of the data can be calculated:

(3) $$R_{L}=\frac{T+G+Tch}{3}$$

(4) $$R_{P}=\frac{Gm+V}{2}\;(option\;A)\;or\;R_{P}=E\;(option\;B)$$

The overall data quality level is determined according to the achieved DQR, following the criteria presented in Table 2. The same criteria apply for reliability and representativeness.

The new framework establishes a clear distinction between the indicators to assess representativeness and those to assess reliability. For the former, temporal, geographical and technological correlations are defined, similar to the ones presented in the studied methods. However, for the latter, two new indicators are established, Generation method and Validity. Previous studies assessed both aspects in one indicator (normally called Reliability), making its implementation difficult in many cases. In addition, indicators related to completeness were removed from the framework. This indicator has been defined to assess the number of sites for which the datum is representative, or data related to the relevant mass flows of the study. Clearly, these aspects are not related to the parameters identified in this study, reason why that type of indicator was not considered.

This framework was developed because the existing methods for quality assessment were not suitable for the assessment of the collected data. The main reason is that the methods (excluding the one developed by Laner and colleagues) were established for the assessment of life cycle inventory datasets, which are related to LCA aspects (e.g. Manfredi and colleagues defined the indicators based on the coverage of environmental footprint impact categories, and on methodological choices such as allocation and substitution). The method by Laner et al. (2015a) suited better the data to evaluate, but still did not fully tackled all the aspects related to their quality, e.g. the indicator Completeness is clearly only applicable to mass flows.

It is important to indicate that the methods presented by Weidema and Wesnæs, and Laner and colleagues were developed not only to assess data quality, but as a basis to estimate the uncertainty of the data. They proposed that, based on the DQIs score and assuming a specific probability distribution, coefficients of variation (CVs) can be estimated. The shortcoming of this approach is that the calculation of the CV is based on subjective criteria. On the one hand, it depends on the definition of each indicator, for example, the temporal correlation can be defined every 10 years, 5 years, 3 years, or any number of years the researcher proposes. On the other hand, it depends on the scoring of each indicator. There is already evidence that LCA practitioners, even with a similar level of experience, show a very poor consistency at scoring existing Pedigree-matrix systems (Edelen and Ingwersen, 2016). In 2007, Lloyd and Ries (2007) studied quantitative approaches to assess uncertainty in LCA, and recommended caution in aggregating DQIs and translating them directly into distributions for propagating uncertainty.

Considering this, it was chosen to apply DQA as a qualitative approach to assess data uncertainty, similar to those proposed by BIO by Deloitte (2015); Edelen and Ingwersen (2016), and Manfredi et al. (2012). Even though the method exhibits the same subjective aspects mentioned before, there is no mathematical meaning of the final DQR. Here, the DQR serves to make the quality of information more transparent, to simplify the literature review process, to identify potential data quality issues in large datasets, and to select data from databases.

The proposed framework can be applied in MFA and in CA. In the case of CA, the framework represents a more advanced method compared to the existing ones, assessing different aspects of the data. In practice, this framework can help in: (i) identifying the materials that require more representative and/or reliable data for their assessment, and (ii) showing how reliable the criticality calculation is, especially for the materials that are close to the threshold of the criticality zone. A set of colours could be used for this, to distinguish reliable results from less reliable results.

### 3.3 Case study: Overall data and source analysis

Throughout the literature research, more than 330 sources were consulted for the collection of data related to Co. Data were acquired from 76 sources, and a total of 302 values were gathered, while harvesting data for 160 parameters. These data are related to different part of the world, covering from the 70 s to the present.

**Fig. 2:**
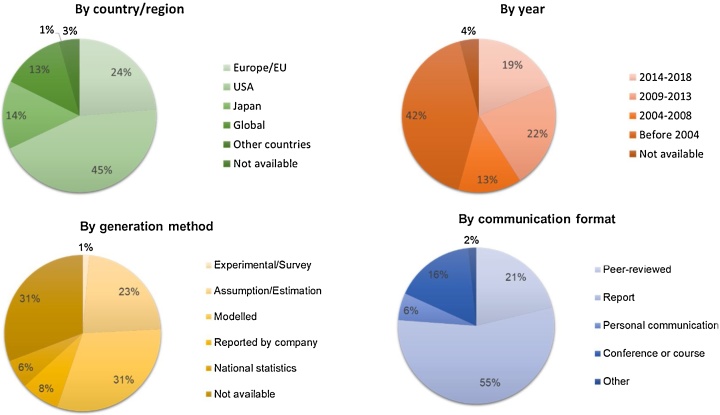
Analysis of available data relevant to model the flow of Co throughout the technosphere. Represented according to covered country/region, year(s), generation method, and communication format.

The analysis of the data was developed according to the criteria described in Section 2.4. Fig. 2 depicts the analysis performed according to the covered country/region, the year(s), the data generation method, and the communication format. From a geographical perspective, 83% of the values are associated to the USA, Europe/EU and Japan. Data related to the USA represents 45%, followed by Europe/EU with 24%. Visibly, most of the data come from developed economies. Regarding the temporal representation, 54% of the data cover the last 15 years (2004–2018), and 42% consist of older data. However, data from the last 5 years only represent 19% of the total available data. Related to the generation method, it is observed that modelled or calculated values represent around one third of the total data. Data for which the generation method is not clear represent another 31%. Remarkably, 23% of the data consist of mere assumptions or rough estimations. In addition, data generated by statistical offices and reported by companies only represent 14% of the total data. Concerning the Communication format, more than 90% of the data come from peer-reviewed articles, reports and conferences or courses. Data from reports cover 55% of the total data. Most of the consulted reports correspond to reports commissioned by governmental institutions, such as the Joint Research Centre (JRC) of the European Commission, and the United States Geological Survey (USGS).

**Fig. 3:**
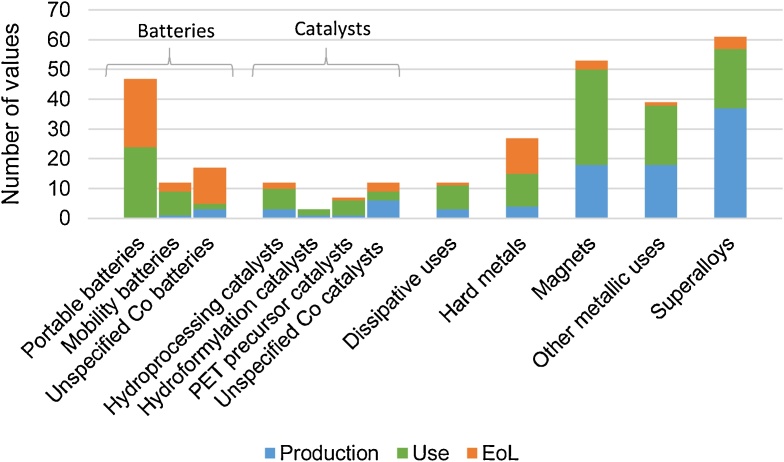
Number of reported values, represented by application and life cycle phase.

Fig. 3 shows the analysis of the number of reported values, per application and per life cycle phase. It is observed that superalloys is the application with the highest number of collected values (61), followed by magnets (53) and portable batteries (47). The applications with the lowest number of values are catalysts (12, 3, 7, and 12 values for hydroprocessing, hydroformylation, PET precursor production, and unspecified Co catalysts, respectively), mobility batteries (12), and dissipative uses (12). From the life cycle perspective, most of the values are associated to the phases Production and Use, with the exception of batteries, for which most of the data are related to Use and EoL.

Already in the 1980s, Co was recognized by the USA as a critical material in industrial and military applications, commissioning a number of studies to address their production and recycling (National Research Council, 1983; OTA, 1985; Shedd, 1993). In those years, 74% of the consumed Co was used in the production of alloys. For this reason, special attention was given in these reports to alloy-related products, such as superalloys, magnets and other metallic uses. Most of the gathered values related to the production of these applications come from these studies, which explains the high number of data related to the Production phase, and the high representation of the USA and data from before 2004.

For the EU, most of the data are related to the Use and EoL phases of waste electrical and electronic equipment (WEEE), and portable batteries. Since 2000s, the EU has been one of the main actors on WEEE recycling, together with the USA, Japan, and China (Melin, 2018; Zeng et al., 2018). WEEE and Battery directives entered into force in 2003 and 2006, respectively, in order to develop schemes that arrange collection and further transportation of waste to recycling facilities (European Commission, 2018a, e). In line with these directives, studies and projects like ProSum were developed, with the purpose to provide central access to data related to secondary resources from “urban mines” (Huisman et al., 2017).

The information from Japan is mostly composed by values related to the Use phase. In this country, different studies have been performed to assess the lifetime and the lifetime distribution of a number of products. These studies cover products concerning the applications superalloys, magnets and other metallic uses; such as engines, turbines, heat exchanger, electric motors, PCBs and tools steels.

Even though the focus of the research was to collect data and information for Europe, it is noteworthy that no data are presented for China (compared to other non-European countries such as the USA and Japan), although this country is the main refiner of Co in the world, with a main role in battery recycling (Darton Commodities Limited, 2018). The two presumed reasons for this are language limitations and data confidentiality, which impedes the availability of information from China in the literature.

The lack of data from China is potentially one of the reasons for the low number of values related to the production and use of mobility batteries. China is the main producer of Li-ion batteries used in hybrid and electric cars (xEV), where half of global xEV sales takes place (Darton Commodities Limited, 2018). xEV vehicles are relatively new in the market, which explains the low number of values related to the EoL phase. Before 2010, Li-ion batteries were principally found in portable electronic products, until the first electric cars were launched and plug-in hybrid electric vehicles with lithium-ion batteries started to appear. Considering that these vehicles have lifetimes of about 10 years, it is expected that only around 2020 a significant number of mobility batteries will reach their EoL (Darton Commodities Limited, 2018; Melin, 2018).

To finalize this section, some words should be said about the evaluated parameters. As it was established in Section 2.1, the selection of parameters was made in order to obtain the necessary data for estimating material flows in the technosphere (in this case Co flows), reason why various parameters are closely related or complementary. For example, the parameters of the Production phase (yield, scrap recovery percentage, and scrap downcycling percentage) are linked through the losses. If the value of the three parameters are known, the losses of the phase can be estimated. Unfortunately, these values were found only for a few applications, which makes necessary establishing assumptions in order to perform the calculations. In the case of the Use phase, lifetime and hoarding time values can be overlapped. Lifetime can include a single life (before the collection or disposal) or multiple lives through reuse, repair and refurbishment. Moreover, these multiple lives can be (or not) separated by hoarding periods. However, for many of the found values, the source do not specify which period is considered as lifetime of the application. Related to the Use and EoL phases, the percentages of hoarding, collection and disposal are connected. Products at EoL can follow different paths: hoarded by users, collected for recycling, disposed in waste bins (to be afterwards landfilled or incinerated), reused or refurbished for a second life, or can follow unidentified streams due to unreported reuse, recycling and various trade and export informal channels (Huisman et al., 2017; thinkstep, 2017t; Melin, 2018). However, most of the available data only address the first three paths, and some sources consider that if two of the parameters are known the remaining one can be estimated.

### 3.4 Data quality analysis per application

The analysis of the data and further application of the DQA framework allowed selecting the values with the highest quality. This was done considering the representativeness of the data for the EU at present. The selected values, together with their overall quality level (presented by colours) are shown in Table 3 (the complete dataset with all the values and the DQR, R_L_, and R_P_ scorings is in SI). To select the data, the DQR of the values of each parameter were compared, selecting those with the lowest DQR (highest quality level). In case no data was reported, a red question mark is used; when a parameter does not apply to the application, NA (not applicable) is used.

## Batteries

Portable batteries show well-documented Use and EoL phases. The production of this application was not addressed, as Europe does not produce this kind of battery at present (EBRA, 2018). Data were found for almost every parameter, except for the distribution to recycling processes (11% gap, unavailable data for 1 out of 9 parameters). In total, 47 values were found with quality ranging from Very high to Very low. Nevertheless, most of the data present High or Low quality.

By the application of the DQA framework, 18 of the 47 values were selected for the final dataset. The quality level of these values is Very high or High. In average, these values present Very high representativeness and Low reliability.

It is observed that the highest quality value for lifetime is 6 years, which includes the hoarding period. However, the overall found values extent from 1 to 10 years. There are different reasons to explain this variation. First, many of these values are not specific for the battery itself, but for the device in which the battery is embedded. Cell phones, laptops, digital cameras, and other household products use rechargeable batteries for their functioning, each one having different periods of use. Second, some of the values are indicated as first life, others consider the hoarding time, and others consider first life, hoarding time and second life of the battery. It is noteworthy that in a high number of cases, the source does not specify which type of lifetime is addressed.

There is also a significant difference between the values reported for EoL products misplaced in waste bins (non-selective collection rate) and for collection rate. For the former, the reported values (all of them presenting High quality) are 2.3% or between 19 and 29%. For the latter, the values with the highest quality are between 5 and 17%. However, the range considering all the values goes from 3 to 50%. Although a discrepancy was expected due to data from different countries/regions and different years, this discrepancy is still observed in data for Europe considering the last 5 years. One of the reasons that explain this difference is the type of device for which the data is representative. Some of these numbers are specific for cell phones or laptops, others for household products, and others for Li-ion batteries. Another plausible explanation is the lack of harmonization of the reporting of volumes of collected waste batteries and WEEE, fact already indicated in previous research (Huisman et al., 2017).

EoL devices are significantly hoarded by users before (1) the consumer brings them to a collection system or (2) are dispose of as municipal waste, which agrees with low collection rates. The high hoarding percentage and the low collection rate were corroborated by the consulted expert (EBRA, 2018). In addition, the expert indicated that portable Li-ion batteries have longer service life, more than 10 years in some cases.

For mobility batteries, 12 values were found for 5 out of 15 parameters (67% gap). The quality of these values range from Very high to Low. By the DQA, 5 of the 12 values were selected for the final dataset, with Very high or High quality level. These data show in average Very high representativeness but Very low reliability.

The literature research provided little information about the production of this application. Furthermore, no data were found for the EU or Europe. The reason of this is that currently in Europe there is no massive production of Li-ion batteries for mobility. However, this should change in the coming years due to the recently launched EU Battery Alliance, which attempts to establish a competitive manufacturing value chain of mobility batteries in Europe (European Commission, 2018c).

Visibly, the Use phase is the most well-documented. A single life of this application is regarded as 10 years by different sources. However, several studies indicate that EoL batteries used in xEV vehicles and industry, could be reused in off-grid and grid-based stationary energy storage applications (EPA, 2013; Olofsson and Romare, 2013; Richa et al., 2014; Darton Commodities Limited, 2018; Melin, 2018), for a second life of 4–10 years (Ahmadi et al., 2017; Bobba et al., 2018; Saubermacher Dienstleistungs AG, 2018). The refurbishment of xEV Li-ion batteries is still in pilot stage, since collection of this type of battery will massively take place only after 2020 (EPA, 2013; Darton Commodities Limited, 2018).

The category of unspecified Co batteries is better documented than the previous one; 17 values were found for 8 of 15 parameters (47% gap). Ten of these values were selected for the final dataset, which show a quality level ranging from Very high to Low, and an average High representativeness and Very low reliability.

Opposite to portable and mobility batteries, values related to production were found for this application. The values found for the Use phase are more similar to those found for mobility batteries; and the ones of the EoL phase, more similar to those of portable batteries. According to literature, the recycling efficiency of this application ranges between 65 and 98%; nevertheless, the values with the highest quality indicate that the efficiency varies between 90 and 98%.

It is important to point out that mobility batteries are composed by identical cells to those used in portable devices, which are produced and recycled through the same processes (EBRA, 2018). This is the reason why a significant number of values were found for unspecified Co batteries, without any specification about the addressed type of battery. Several of the values contained in this category are valid for portable and mobility batteries, complementing their datasets.

## Catalysts

Clearly, the three specific type of catalysts are poorly documented, being hydroformylation catalysts the one with the lowest data availability (82% gap, 9 out of 11 parameters not available). Overall, 22 values were found, with quality ranging from Very high to Very low. Nonetheless, the majority of the values present Low quality.

Through the DQA, 20 of the 22 values were selected. The quality level varies depending on the type of catalyst; for hydroprocessing catalysts the common level is Low quality, for hydroformylation catalysts High and Low quality, and for PET precursor catalysts from Very high to Low quality. The values with the highest quality are related to the catalysts used in the production of PET precursors, which were obtained through personal communication. In average, the values for the three type of catalysts present Very low reliability and High representativeness.

It is possible to observe that the Weibull distribution parameter does not apply to any catalyst. This lifetime distribution modelling has been widely used to simulate product lifetimes (Davis et al., 2007), but no evidence was found about its application on catalysts (Khorashadizadeh and Atashi (2017) used the Weibull distribution to model the kinetics of hydroformylation catalysts, but not the lifetime).

According to different sources, EoL hydroprocessing catalysts can be recycled for Co recovery, or downcycled for steel production (National Research Council, 1983; Marafi and Stanislaus, 2008; Akcil et al., 2015). Catalysts used in the plastic industry are indirectly recycled to the same process, due to the recycling of PET bottles (Committee of PET Manufacturers in Europe, 2018). For hydroformylation catalysts, no data were available regarding recycling.

Like in the case of batteries, more data are available for unspecified Co catalysts. In total, 12 values were found, with a quality range from High to Very low. By quality assessment, 10 values were selected for the final dataset, with High or Low quality level. As for the specific type of catalysts, the data present Very low reliability but High representativeness. The collected data could be used as approximated data for the specific catalysts, in order to complement their datasets.

## Dissipative uses

This application is well-documented, with available information for 10 of the 12 parameters that apply to it (17% gap). Twelve values were found, most of them showing Low quality. The quality assessment left no value out of the dataset. In average, these values present Very low reliability and High representativeness.

It is observed that for lifetime, the gathered values are 1 year or from 5 to 25 years, which is explained due to the diversity of products that are considered in this category. Clearly, these products are not recycled at their EoL, reason why the parameters related to recycling are not applicable.

## Hard metals

Hard metals is one the most well-documented applications of Co, together with portable batteries and superalloys. Twenty-seven values were found for 13 of the 15 parameters (13% gap), which are characterised by High or Low quality.

By the quality assessment, 25 values were selected. The data quality varies from Very high to Low, being High quality the predominant level. In average, these data present Very low reliability and High representativeness.

According to the literature research, there is a significant difference between the values for lifetime: 2 or 11 years. According to experts, around 90% of hard metals used at industrial scale are returned to manufacturing facilities to be retooled (Cobalt Institute, 2018), which explains a long lifetime. However, a lifetime of 11 years was regarded as too long. Although it depends on the application, a weighted average should be lower according to an industrial expert (see SI). Noticeably, this application is highly hoarded by users at its end of use, but for a short period. A plausible explanation is that industrial hard metals are accumulated during the hoarding period, to be later recycled in a larger volume. The non-selective collection rate also shows a high value, while collection rate varies from 15 to 75%. One of the consulted industrial experts indicated that not all collected hard metals are recycled to recover Co, but a large part is downcycled to produce steel (see SI), which can explain the significant variation of this parameter.

For pre-treatment, two values were found: 77%, and almost 100%. The former corresponds to sorting efficiency and the latter to the efficiency of a chemical process to separate Co from tungsten.

Hard metals are industrially recycled by more than one route (mainly by the Zn process and chemical processes). The degree of contamination of the scrap will determine which recycling method applies (National Research Council, 1983). The recycling distribution values indicate that chemical methods are used in a larger extent than the Zn method. The scrap from chemical recycling processes is downcycled to steel production, or even reused for carbide production (Personal communication with industrial expert, see SI).

## Magnets

Magnets and magnet-embedded devices are fairly documented, with 53 values for 10 of the 13 applicable parameters (23% gap). The data present High or Low quality, being High the predominant level.

Thirty-six values were selected after the application of the DQA framework. The common level of these values is High quality, with in average Low reliability and High representativeness.

Considering the overall data, significant differences are found in the values of the manufacturing yield (50–98%), the lifetime (5–22 years), and the collection rate (10–90%). After the quality assessment, these values were reduced to 94–98%, 7–16 years, and 35 or 45%, respectively.

Lifetime values are not related to magnets as a product, but to the devices that contain them. This applies in general to the information related to the Use and EoL phases of magnets, for which data about electric motors (industrial and mobility), generators, and disk drives were considered.

Noticeably, parameters related to recycling do not apply to this application. Several of the consulted documents indicated that there is no information of any current activity in the post-consumer recycling of magnets on a large scale, and that limited attention has been given to the recovery of metal values from discarded magnets. If recycling takes place, Co is not recovered but used in steel production. Most possibly, this lack of attention is because Co magnets represent a small percentage of the magnet market and do not possess magnetic properties that are as good as other permanent magnets, such as NdFeB magnets (Liu and Chinnasamy, 2012; Tunsu et al., 2015; Rainer, 2016; Sinha et al., 2017). Different experts confirmed this information for the USA and the EU (BRGM, 2018; CMI, 2018; UK Magnetics Society, 2018).

## Other metallic uses

For this application, 39 values were found for 10 of the 13 applicable parameters (23% gap). The quality ranges from High to Very low, with the majority of the values presenting Low quality.

After the assessment of the quality, 24 values were selected. Forty percent of the parameters present High quality level, and 60% Low quality level. In average, the data show Low reliability and Low representativeness.

From an overall perspective, lifetime and collection rate present values with significant differences. For the lifetime, values between 5 and 13 years were found; for the collection rate, values between 15 and 40%. By the application of the DQA framework, these values were restricted to 11 years, and 35%, respectively.

As in the case of magnets, other metallic uses consist of a number of different devices. Tool steel, hardfacing alloys, and semiconductors (PCBs and related products) were included in this category. Most of the values of the parameters comprised in Production are related to the former two. In turn, the values for Use and EoL phases are representative for the latter.

Nowadays Co is not recovered from recycling hardfacing alloys or semiconductor-embedded products. According to literature, the recycling of PCBs is rather limited, and between 70 and 80% are landfilled or incinerated at their EoL. As for magnets, if recycling takes place Co is not recovered but used for steel production. It is argued that recycling strategies are not implemented yet due to the requirement of highly sophisticated processes for their recovery (Goosey and Kellner, 2002; Ghosh et al., 2015; Kaya, 2016; Ueberschaar et al., 2017). In the case of tool steels, no information was found in literature about their recycling. However, according to experts, in Germany tool steels are collected and sent to recycling facilities, where Co is recovered (DERA, 2018).

## Superalloys

This application is the most complete, with 61 available values for 14 of the 15 parameters (7% gap). The quality ranges from High to Very low. Nonetheless, the predominant levels are High and Low.

By the application of the quality assessment, 35 of the 61 collected values were selected for the final dataset. The selected data present High quality for the majority of the parameters. In average, the reliability is Low but the representativeness is High.

The gathered data are mostly related to superalloys used in turbine blades and jet/rocket engines, heat exchanger tubing, and industrial gas turbines; which explains the large variation of some of the parameters.

The insight of the consulted experts confirmed the collected data. According to them, the quality of superalloys and magnets is extremely important, reason why 40–50% becomes scrap during their production (Kanva EU Ltd, 2018). Moreover, production scrap is more recovered than post-consumer waste, because the latter is made of several alloys, which makes its recycling more difficult (BRGM, 2018). According to literature, of the collected post-consumer waste, 59 or 63% is recycled for metal recovery, and 37 or 41% is downcycled for steel production. One of the consulted experts (​Glencore Nikkelverk, 2018​) indicated that these numbers vary, depending on the value of Co in the market.

### 3.5 General perspective

For the case study of Co, a total of 302 values were gathered for 98 of the 160 parameters; data were unavailable for 39% of the parameters, which were not possible to obtain from literature or through personal communication. The most common data quality levels for the complete dataset are High and Low. The biggest data gaps are for the three types of catalysts (hydroprocessing 64% gap, hydroformylation 82% gap, PET precursors 71% gap) and mobility batteries (67% gap). Regarding the life cycle phases, the biggest gaps are observed in EoL (42% gap), followed by Production (41% gap), and Use (32% gap). The parameters with the lowest number of values are the distribution to recycling processes (67% gap), and the processing scrap recovery, processing downcycled scrap, and pre-treatment efficiency (64% gap for each one). The final dataset (built after the quality assessment) is composed by 195 selected values; 35% of the collected values were left out due to their low quality. Seven percent of the parameters present Very high quality, 51% High quality, and 42% Low quality. None of the values presents Very low quality.

The application of the DQA framework allowed analysing the data regarding its representativeness and reliability. For the study case, the data show in general a higher representativeness than reliability. The low reliability is because the data generation methodology is unknown for the majority of the collected values. In addition, a significant number of values are assumptions or estimates, or are obtained from one or two stakeholders.

Finally, from the results shown in Table 3, it is clear that many assumptions will be required to calculate the flows of Co in the technosphere. However, certain parameters are regarded as more critical than others. For instance, the information about the production of mobility batteries is key, since this application entails the main consumption of Co in the world (Darton Commodities Limited, 2018). Although the data collected for unspecified Co batteries could be used to fill these gaps, this is not possible for every parameter. It is also possible to state a difference between hoarding periods by costumers and by industries. The former is likely more critical than the latter, because industries presumably hoard EoS products for less time than costumers do. Another example is the general lack of information about pre-treatment efficiency and distribution to recycling processes, which produces low reliable calculations of flows related to the EoL phase.

## 4 Conclusions

The present research contributes to a better understanding of the societal metabolism of Critical Raw Materials (CRMs), with a special focus on secondary resources. The developed flowchart and the identification of the key parameters of each phase of the life cycle allowed defining what is needed to assess and estimate CRMs flows in the technosphere. The scheme can be used as a basis to improve data production, collection and use, not only by researchers of the field but also by industries and policy makers.

Moreover, the study also includes the development of a Data Quality Assessment (DQA) framework for Criticality Assessment (CA), and Material Flow Analysis (MFA). The method improves the assessment of reliability from previous literature, introducing the distinction between the Generation method and the Validity of the datum. Furthermore, it defines an advanced method applicable to CA, which was not available to date. The method does not estimate quantitatively the uncertainty of the data, but it contributes to more transparent results, indicating where and how to improve the data, making the distinction in terms of representativeness and reliability. Depending on which aspect has to be improved, specific stakeholders could be consulted.

Finally, a case study was developed for cobalt (Co). Its main applications were better identified and detailed, and data was collected and analysed for the key parameters established in the flowchart. The case study on Co shows the usefulness of the schemes, which could be applied to any CRM. Similar studies could be developed using these schemes to establish the availability of the data required for CA (in the EU or in any other region or country), indicating data gaps, which phases are well-documented, and which ones require more data. However, such studies are very intensive in time consumption. Focusing only on the most relevant applications of each materials might help saving time. It is also recommended to prioritize the analysis of the materials with the highest criticality, to have more reliable and robust results for the most important materials.

It is concluded that together with data gaps filling, the main challenge related to data for Co is their reliability. To obtain reliable results about Co flows, the methods of the data generation should be more transparent, involving a wider range of stakeholders to assist in filling gaps. Priority should be given to crucial parameters such as the processing and manufacturing yields of mobility batteries, and the pre-treatment efficiency of several applications.

The developed schemes and their application in the Co case are a significant contribution to the study fields of MFA, CA and CRMs, helping to better address and assess the underpinning data of such studies that have an impact in raw materials research and policy.

## Declaration of Competing Interest

The authors declare that they have no known competing financial interests or personal relationships that could have appeared to influence the work reported in this paper.
