# Supplementary material for: Data quality assessment framework for critical raw materials. The case of cobalt
Source: Resour Conserv Recycl. 2020 Jun;157:104564. doi: 10.1016/j.resconrec.2019.104564 (PMC7224515; doi:10.1016/j.resconrec.2019.104564)
Supplement: Supplementary file 1 [file mmc1.pdf]

DATA QUALITY ASSESSMENT FRAMEWORK FOR CRITICAL RAW MATERIALS

THE CASE OF COBALT

María Fernanda Godoy León, Jo Dewulf

Research Group STEN, Ghent University, Belgium

SUPPLEMENTARY INFORMATION

1. Applications

The description given in this section is specific for the applications studied in the research.

Portable batteries

Portable batteries are defined as sealed batteries that can be carried by hand, excluding industrial and vehicle batteries (European Commission, 2006). Cobalt is found in lithium-ion (Li-ion), nickel-cadmium (NiCd) and nickel-metal hydride (NiMH) batteries, being Li-ion batteries the most commonly used (Cobalt Institute, 2018). Li-ion batteries based on lithium cobalt oxide ($LiCoO_2$, LCO) present the higher amount of cobalt (approximately 14%), while in NiCd, NiMH and in Li-ion batteries based on lithium nickel manganese cobalt oxide (NMC) and lithium nickel cobalt aluminum oxide (NCA), the metal is present only in minor amounts (Sommer et al., 2015). The three type of batteries are rechargeable, and can be found in mobile phones, laptops, tablets, cameras, cutting tools, and other household products.

In 2017, around 23% of the global consumption of Co was in portable batteries (Darton Commodities Limited, 2018).

Mobility batteries

Mobility batteries are found in electric vehicles and hybrid electric vehicles (xEV), electric trains, electric buses, and electric bikes (Cobalt Institute, 2018). The battery stores electrical energy that the electric motor uses to power the vehicle (European Environment Agency, 2016). Batteries containing cobalt used in these devices are NMC, NCA, and NiHM batteries (Al-Thyabat et al., 2013; Darton Commodities Limited, 2018).

In 2017, 29% of the global consumption of Co was in mobility batteries. Around 1% was used in ESS (energy storage systems) (Darton Commodities Limited, 2018).

Hydroprocessing catalyst

Hydroprocessing catalysts are used to produce clean fuels and other usable oil feedstocks and products, upgrading oil fraction through the removal of impurities such as sulphur and nitrogen. By adding hydrogen, they also improve the properties and performance of the products (Darton Commodities Limited, 2018). The activity of the catalyst decreases in time, and when it drops below an economically determined level, the catalyst is removed from the reactor. The three main reasons for the loss of activity are coke deposition, poisoning, and sintering of the active phase. Depending on the type of deactivation, the catalyst can be regenerated, recycled, or sent out for disposal (Dufresne, 2007).

In 2017, 3% of the global consumption of Co was in hydroprocessing catalysts (Darton Commodities Limited, 2018).

Hydroformylation and GTL process catalysts

Cobalt catalysts are used in the production of different hydrocarbons, through the hydroformylation reaction and the gas to liquid (GTL) process. In hydroformylation, an alkene reacts with CO and H_2 to produce aldehydes, which are then used in the production of alcohols (used in detergents and plasticizers), and carboxylic acids (used in pharmaceuticals) (De et al., 2013). The GTL process is commonly performed using Fischer–Tropsch synthesis (FTS), where syngas (CO and H_2) react to produce liquid hydrocarbons, which are further processed into liquid fuels (e.g. gasoline, jet fuel, and diesel) (Dry, 1999).

Hydroformylation uses homogeneous catalysts (same phase as the reactants), while FTS involves heterogeneous catalysts (different phase from the reactants) (De et al., 2013; Jahangiri et al., 2014). In hydroformylation, the catalyst is recycled to the process. However, it tends to build-up on the walls of the reactor and on the tower packing, which makes it necessary to periodically remove it and send it to recycling (National Research Council, 1983; Hebrard and Kalck 2009). In FTS, the catalyst can be regenerated to be used in the same process or recycled for metal recovery (Brumby et al., 2005; Jahangiri et al., 2014).

In 2017, 0.5% of the global consumption of Co was in hydroformylation and GTL process catalysts (Darton Commodities Limited, 2018).

PET precursor catalyst

Cobalt-manganese-bromide (CMB) and cobalt-manganese-acetate (CMA) are liquid catalysts used for the oxidation of para-xylene (PX), in the synthesis of terephthalic acid (TPA) and di-methyl terephthalate (DMT). These compounds are raw materials in the production of polyester fibres articles, such as polyethylene terephthalate (PET) bottles, films, and paints (Joo et al., 2016; Darton Commodities Limited, 2018). CMB and CMA are homogeneous catalysts, which are recycled to the process. (National Research Council, 1983; Joo et al., 2016).

In 2017, 2.6% of the global consumption of Co was in PET precursors catalysts (Darton Commodities Limited, 2018).

Dissipative uses

Dissipative uses include applications of cobalt chemicals, from which the metal is not recovered. This category comprises pigments and drying agents; and agricultural, nutritional, and medical uses. Pigments and drying agents are applied in glasses, ceramics, refractories, driers, paints, and varnishes. In agriculture and nutrition, chemicals are used to correct cobalt deficiencies in soils and in animals. Medical uses includes the treatment of certain types of anemia, and as an antidote in cyanide poisoning (Donaldson and Beyersmann, 2012).

In 2017, around 8% of the global consumption of Co was in dissipative uses (Darton Commodities Limited, 2018).

Hard metals

Hard metals or cemented carbides are composite materials consisting of hard tungsten carbide (WC) particles bonded together by a metallic binder. This metallic binder is usually Co, although it can also be Fe, Ni, and other metallic phases (Freemantle et al., 2014; Cobalt Institute, 2018). Its uses comprises cutting tools and wear-resistant components in metalworking, mining, oil drilling, and construction industries. Cobalt is also used in diamond tools, applied as a binding agent to hold together wear-resistant particles (in this case diamonds) (Shedd, 2004).

The two main methods for the recycling of hard metals are chemical processes and the zinc process (Shedd, 2004). The former considers the use of acids, electrochemistry or chemical modification techniques (Freemantle et al., 2014); the latter consists of the addition of Zn, which dissolves the binder phase of the cemented carbide without changing the composition of the material. Chemical processes produce material equivalent to virgin material, contrary to the Zn process that produces material to be used in the production of new hard metals (Kurylak et al., 2016).

In 2017, 7% of the global consumption of Co was in hard metals (Darton Commodities Limited, 2018).

Magnets

Cobalt is mainly found in permanent magnets, which are magnets with high coercivity that can be permanently magnetised by applying a magnetic field (Cobalt Institute, 2018). There are three type of permanent magnets composed by cobalt: AlNiCo (aluminium- nickel-cobalt), SmCo (samarium-cobalt), and NdFeB (neodymium-iron-boron) magnets. Cobalt is mainly found in SmCo magnets (50-60%), followed by AlNiCo magnets (3-36%) and NdFeB magnets (1-5%) (Donaldson and Beyersmann, 2012; Sinha et al., 2017; Cobalt Institute, 2018). Permanent magnets are applied in automotive (traditional, hybrid, and electric), computer and consumer electronics, wind turbines, HVAC (heating, ventilating and air conditioning), and industrial motors (Benecki, 2017).

In 2017, 3% of the global consumption of Co was in magnets (Darton Commodities Limited, 2018).

Other metallic uses

This category includes semi-conductors and integrated circuits, tool steels, and hardfacing and coatings. Semi-conductors and integrated circuits are contained in all modern electronic devices or systems (e.g. televisions, laptops, cameras, cell phones) (Cobalt Institute, 2018). Tools steel are used to work, cut and form metal components, for which they require high hardness and strength (International Molybdenum Association, 2018). Hardfacing and coating is the process where harder material is put onto to a base metal. It is used to increase the wear resistance of metallic components, or to refurbish a surface that is worn-down on used parts (A&A Coatings, 2018)

In 2017, around 7% of the global consumption of Co was in other metallic uses (Darton Commodities Limited, 2018).

Superalloys

Superalloys are Ni, Fe-Ni, or Co based alloys, usually used at temperatures above 540 ºC (Donachie and Donachie 2002). High melting temperatures, and excellent creep, corrosion, and oxidation resistance characterize this type of material (Srivastava et al., 2014). Due to these properties, superalloys are used in a number of applications such as aircraft, rocket, and gas turbine engines; heat exchanger tubing; and nuclear reactors. Superalloys scrap is partly recycled for Co recovery; the rest is downcycled for steel production (Reck and Graedel, 2012).

In 2017, 16% of the global consumption of Co was in superalloys (Darton Commodities Limited, 2018).

2. Parameters definition

Processing/manufacturing yield

Ratio of usable output from the processing/manufacturing process to the input quantity, expressed as a percentage.

Processing/manufacturing scrap recovery

Ratio of the processing/manufacturing scrap that is recycled to the process to the total scrap produced, express as a percentage.

Processing/manufacturing downcycled scrap

Ratio of the processing/manufacturing scrap that is downcycled (for the production of low-end applications, such as steel) to the total scrap produced, express as a percentage.

Lifetime

Different definitions can be given to lifetime (or lifespan) of a product (National Institute for Environmental Studies, 2018):

Total lifetime: period where the product exists in the society in its original form (regardless of whether the product still functions or not).

Service lifetime: period where the product functions and can be put to use, including the duration of distribution for the next use. Service lifetime differs from total lifetime in that it excludes collection span of discarded products (for recycling or disposal).

Other lifetime-related periods are:

Possession span: denotes how long one owner possesses the product in question.

Duration in use: denotes how long one owner uses the product in question. Duration in use differs from possession span in that it excludes hoarding periods.

Product age: denotes the period from the beginning of life of product to the time of interest.

Shape parameter (Weibull distribution)

The Weibull distribution is a continuous probability distribution, commonly used to assess product reliability and survival analysis, analyse life data, and model failure times. The function is characterised by three parameters: the scale parameter (normally denoted as α), the shape parameter (normally denoted as β), and the location parameter (normally denoted as τ). When τ is zero the function become the two-parameter distribution. The shape parameter determines the appearance or shape of the distribution (Lai et al., 2006).

Hoarding rate

Hoarding or hibernation refers to the dead storage of a product that is no longer in use anymore (Wilson et al., 2017). Here is understood as the hoarding of end-of-service (EoS) products.

This parameter is understood as the ratio of hoarded EoS products to the total EoS products produced in a year, expressed as a percentage.

Hoarding time

Hoarding time refers to the period in which the EoS product is hoarded. It is understood as the time between the EoS of the product until its collection (for recycling or disposal).

Non-selective collection rate

Non-selective collection rate is related to the misplacement of EoL products in waste bins.

This parameter is understood as the ratio of not-selectively collected EoL products to the total EoL products produced in a year, expressed as a percentage.

Collection rate

Waste collection is defined as “collection of solid waste from point of production (residential, industrial commercial, institutional) to the point of treatment or disposal” (Hoornweg and Bhada-Tata, 2012). However, the UNEP defined collection rate as the ratio of EoL products collected for recycling to the total EoL products produced in a defined period (UNEP, 2011). In the case of WEEE, the EU defined it as “the volumes collected divided by the average sum of EEE (electrical and electronic equipment) put on the market in the previous three years” (European Parliament, 2006).

In the research, collection rate is understood as the ratio of EoL products collected for recycling to the total EoL products produced in a year, expressed as a percentage.

It is noteworthy to distinguish between collection rate and recycling rate. The EU and the UNEP define the latter as the collection rate multiplied by the rate of recycling at the treatment facilities, assuming that the total amount of collected WEEE is indeed sent to treatment/recycling facilities (European Parliament, 2006; UNEP, 2011).

Pre-treatment efficiency

Pre-treatment covers several operations such as separation, sorting, physical processes, and chemical processes.

In the research this parameters is understood as the ratio of usable output from any pre-treatment operation (or a set of them) to the input quantity, expressed as a percentage.

Distribution to recycling processes

Waste and scrap can be recycled through different processes. This parameter refers to the distribution of waste or scrap to the different processes (how much of the total waste is recycled by one or another process), expressed as a percentage.

Recycling efficiency

Ratio of usable output from the recycling processes to the input quantity, expressed as a percentage.

3. DQA methods description

In 1996, Weidema and Wesnæs proposed a DQA method called Pedigree-matrix, which consisted of five independent DQIs: Reliability, Completeness, Temporal correlation, Geographical correlation, and Further technological correlation (hereafter referred to as Technological correlation). The indicator Reliability was related to the assessment of the sampling methods and verification procedures. The indicator Completeness defined how complete the available datum was in function of its statistical representativeness, the number of measurements in the sample, and the time periods for data collection. The indicators Temporal and Geographical correlation described the representativeness of the datum regarding its year of generation and the intended geographical area, respectively. The indicator Technological correlation evaluated the congruence of the available data and the targeted data with respect to technology, product, etc. In the matrix, each indicator was described by a score from 1 to 5, with 1 for the highest quality and 5 for the lowest quality. This matrix was established to be used in data quality management for life cycle inventories (LCI).

Manfredi et al. (2012), and Edelen and Ingwersen (2016) established their own modified Pedigree-matrix, to be applied likewise on LCI. Manfredi and colleagues established six DQIs: Completeness, Methodological appropriateness and consistency, Time representativeness, Technological representativeness, Geographical representativeness, and Parameter uncertainty. Edelen and Ingwersen defined DQIs at flow level (making a distinction between reliability and representativeness) and at process level. At flow level five DQIs were given: Reliability, Temporal correlation, Geographical correlation, Technological correlation, and Data collection methods. The latter four were linked to representativeness. For processes, two DQIs were defined: process review and process completeness.

Laner et al. (2015) based their work on the Pedigree-matrix, applying the same five indicators, although modifying their definition to be applicable to MFA studies. Furthermore, an additional DQI was added, termed Expert estimate. This indicator was used alone, as a replacement of the other five. In this work, the DQIs were described by a score from 1 to 4, with 1 for the highest quality and 4 for the lowest quality.

Table A1 present a summary of the comparison between the four described methods.

Table A1. Comparison of available methods for DQA.

Method	Application	Scoring	*Indicators*		
			Reliability	Representativeness	Other
Weidema and Wesnæs (1996)	LCA	1 to 5	Reliability	Temporal correlation<br>Geographical correlation<br>Further technological correlation	Completeness
Manfredi et al. (2012)	LCA	1 to 5	-	Time-related representativeness<br>Geographical representativeness<br>Technological representativeness	Completeness<br>Methodological appropriateness and consistency<br>Precision/uncertainty
Laner et al. (2015)	LCA	1 to 4	Reliability<br>Expert estimate	Temporal correlation<br>Geographical correlation<br>Other correlation	Completeness
Edelen and Ingwersen (2016)	MFA	1 to 5	Flow reliability[a]<br>Process review[b]	Temporal correlation[a]<br>Geographical correlation[a]<br>Technological correlation[a]<br>Data collection methods[a]	Process completeness[b]

a: Flow indicators. b: Process indicators.

4. Full dataset

Table A2. Full dataset of the application portable batteries. It includes the DQA results in terms of R_L (reliability), R_P (representativeness) and DQR (data quality rating).

Parameter	Data country/region	Data year	Value	Unit	DQA R_L	R_P	DQR	Reference
Life time	Global	2001	1-3	years	4.0	2.7	3.2	Contestabile et al. (2001)
Life time	EU 25	2006	5	years	2.5	2.0	2.2	EuP 2007
Life time	EU	2015	6.2	years	2.5	1.0	1.6	Desmet and Colin (2017)
Life time	South Africa	2015	3-5	years	4.0	1.7	2.6	Knights and Saloojee (2015)
Life time	Germany	2012	6.6	years	4.0	2.0	2.8	Buchert et al. (2012)
Life time	Not available	2017	2-4	years	4.0	2.0	2.8	Kumar and Suman (2017)
Life time	EU	2006	2-7	years	4.0	2.3	3.0	Müller and Friedrich (2006)
Life time	Not available	2002	2, 7	years	4.0	3.0	3.2	Heegn et al. (2003)
Life time	EU	2015	7.1	years	2.5	1.7	2.0	Desmet and Colin (2017)
Life time	Japan	2003	4.3	years	3.0	2.7	2.8	Oguchi et al. (2006)
Life time	EU5	2015	1.7	years	4.0	1.3	2.4	Kantar WorldPanel (2016)
Life time	USA and others	2005	2.5	years	4.0	2.3	3.0	Harper et al. (2011)
Life time	Japan	2012	7.4	years	3.0	2.0	2.4	Nomura and Suga (2013)
Life time	Japan	2012	10.2	years	3.0	2.0	2.4	Nomura and Suga (2013)
Life time	EU	2018	>10	years	4.0	1.0	1.8	EBRA (2018)
Shape parameter (Weibull distribution)	Japan	2003	3.1	-	3.0	2.7	2.8	Oguchi et al. (2006)
Shape parameter (Weibull distribution)	Japan	2012	2.57	-	3.0	2.0	2.4	Nomura and Suga (2013)
Shape parameter (Weibull distribution)	Japan	2012	1.61	-	3.0	2.0	2.4	Nomura and Suga (2013)
Hoarding rate	Japan	2010	54.5	% (w)	3.0	1.7	2.2	Asari and Sakai (2013)
Hoarding rate	EU	2006	65	%	4.0	2.3	3.0	Müller and Friedrich (2006)
Hoarding rate	EU	2012	50	% (w)	4.0	1.3	2.0	BIO by Deloitte (2015)
Hoarding time	EU	2012	3.5	years	3.0	1.3	1.8	BIO by Deloitte (2015)
Hoarding time	EU	2006	2-4	years	4.0	2.7	3.2	Müller and Friedrich (2006)
Non-selective collection rate	EU 28 + Switzerland and Norway	2015	18.5	% (w)	2.5	2.0	2.2	Huisman et al. (2017)
Non-selective collection rate	EU 28 + Switzerland and Norway	2015	2.3	% (w)	2.5	2.0	2.2	Huisman et al. (2017)
Non-selective collection rate	Global	2017	19-29	% (w)	2.5	2.0	2.2	thinkstep AG (2017)
Collection rate	EU	2007	2.7	%	4.0	1.7	2.6	Weyhe (2008)
Collection rate	EU	2007	9	%	4.0	2.0	2.8	Weyhe (2008)
Collection rate	EU 28 + Switzerland and Norway	2015	13	% (w)	2.5	1.3	1.8	Huisman et al. (2017)
Collection rate	EU 28 + Switzerland and Norway	2015	6.5	% (w)	2.5	1.3	1.8	Huisman et al. (2017)
Collection rate	EU	2016	5	%	3.5	1.0	2.0	Darton Commodities Limited (2018)
Collection rate	EU	2014	10	%	4.0	1.0	2.2	Circular Energy Storage (2018)
Collection rate	Japan	2010	9	% (w)	3.0	1.7	2.2	Asari and Sakai (2013)
Collection rate	Germany	2011	17	% (w)	3.0	2.0	2.4	Sommer et al. (2015)
Collection rate	Germany	2012	50	% (w)	4.0	2.0	2.8	Buchert et al. (2012)
Collection rate	Germany	2012	5	% (w)	4.0	2.0	2.8	Buchert et al. (2012)
Collection rate	USA	2005	10	%	3.5	2.0	2.6	Wilburn (2008)
Collection rate	Japan	2005	30	% (w)	4.0	2.0	2.5	Harper et al. (2011)
Collection rate	Europe	2017	45	% (w)	2.5	1.3	1.8	thinkstep AG (2017)
Collection rate	EU	2012	64	% (w)	2.5	1.7	2.0	BIO by Deloitte (2015)
Pre-treatment efficiency	Belgium	2013	98	%	3.5	2.7	3.0	Tran et al. (2017)
Pre-treatment efficiency	Germany	2011	95	% (w)	3.0	2.0	2.4	Sommer et al. (2015)
Pre-treatment efficiency	Germany	2012	80	% (w)	4.0	2.0	2.5	Buchert et al. (2012)
Pre-treatment efficiency	EU	2018	97-98	% (w)	3.0	2.0	2.3	Saubermacher AG (2018)
Recycling efficiency	Germany	2011	90	% (w)	3.0	2.0	2.4	Sommer et al. (2015)
Recycling efficiency	Germany	2012	96	% (w)	4.0	2.0	2.5	Buchert et al. (2012)
Recycling efficiency	India	2017	95	% (w)	4.0	1.7	2.6	Kumar and Suman (2017)

Table A3. Full dataset of the application mobility batteries. It includes the DQA results in terms of R_L (reliability), R_P (representativeness) and DQR (data quality rating).

Parameter	Data country/region	Data year	Value	Unit	DQA R_L	DQA R_P	DQA DQR	Reference
Processing yield	USA	2017	85	%	4.0	1.3	2.4	Dai et al. (2017)
Life time	USA	2011	10	years	4.0	2.0	2.8	Environmental Protection Agency (2013)
Life time	USA and others	2005	8	years	4.0	2.3	3.0	Harper et al. (2011)
Life time	EU	2014	10	years	3.0	1.0	1.8	EUROBAT (2014)
Life time	Global	2015	8	years	3.0	1.7	2.0	Ahmadi et al. (2017)
Life time	Global	2010	10	years	4.0	2.0	2.5	Ziemann et al. (2018)
Life time	Global	2014	10	years	4.0	2.0	2.5	Habib and Wenzel (2014)
Life time	Global	2016	8-10	years	4.0	1.7	2.6	Jiao and Evans (2018)
Hoarding rate	EU	2012	0	% (w)	4.0	1.3	2.0	BIO by Deloitte (2015)
Collection rate	EU	2012	100	% (w)	4.0	1.3	2.0	BIO by Deloitte (2015)
Collection rate	Global	2005	90	% (w)	4.0	2.7	3.0	Harper et al. (2011)
Collection rate	Europe	2017	95	% (w)	2.5	1.0	1.6	thinkstep AG (2017)

Table A4. Full dataset of the application unspecified Co batteries. It includes the DQA results in terms of R_L (reliability), R_P (representativeness) and DQR (data quality rating).

Parameter	Data country/region	Data year	Value	Unit	DQA R_L	DQA R_P	DQA DQR	Reference
Manufacturing yield	Belgium and others	2008	100	% (w)	3.0	2.0	2.4	Dewulf et al. (2010)
Manufacturing yield	USA	1980	92	% (w)	2.5	2.7	2.6	National Research Council (1983)
Production scrap recovery	USA and others	2005	100	% (w)	4.0	1.7	2.3	Harper et al. (2011)
Life time	Japan	2002	10.9	years	3.0	2.7	2.8	Nomura (2005)
Shape parameter (Weibull distribution)	Japan	2002	2.2	-	3.0	2.7	2.8	Nomura (2005)
Collection rate	Europe	2018	10.0	%	4.0	1.0	2.2	Tytgat and Van Damme (2018)
Pre-treatment efficiency	EU	2018	80-90	% (w)	3.0	1.0	1.5	Undisclosed company
Recycling efficiency	EU	2010	>98	% (w)	3.5	1.3	2.2	Swart et al. (2014)
Recycling efficiency	EU	2004	70	% (w)	4.0	1.7	2.6	Georgi-Maschler et al. (2012)
Recycling efficiency	EU	2009	65-80	% (w)	4.0	1.7	2.6	Meskers et al. (2009)
Recycling efficiency	Not available	2004	80	% (w)	4.0	2.3	3.0	Saeki et al. (2004)
Recycling efficiency	EU	2017	90	%	4.0	1.3	2.4	Mathieux et al. (2017)
Recycling efficiency	EU	2012	85	% (w)	4.0	2.0	2.5	BIO by Deloitte (2015)
Recycling efficiency	EU	2017	For different type of cells	kg/kg of cell	2.5	1.0	1.6	thinkstep AG (2017)
Recycling efficiency	Not available	Not available	94	% (w)	4.0	2.7	3.2	Lebedeva et al. (2016)
Recycling efficiency	Global	Not available	>90	%	3.5	1.7	2.4	Kushnir (2015)
Recycling efficiency	Global	Not available	>95	%	3.5	1.7	2.4	Kushnir (2015)

Table A5. Full dataset of the application hydroprocessing catalysts. It includes the DQA results in terms of R_L (reliability), R_P (representativeness) and DQR (data quality rating).

Parameter	Data country/region	Data year	Value	Unit	DQA R_L	DQA R_P	DQA DQR	Reference
Processing yield	USA	1980	97	% (w)	3.5	2.7	3.0	Shedd (1993)
Manufacturing yield	USA	1980	96	% (w)	3.5	2.7	3.0	Shedd (1993)
Life time	USA	1980	0.5-several	years	3.5	2.3	2.8	National Research Council (1983)
Life time	USA	2005	2	years	4.0	2.0	2.5	Harper et al. (2011)
Regeneration times	Europe	1994	2-3	times	4.0	2.3	3.0	Berrebi et al. (1994)
Regeneration period	Europe	1994	2-6	years	4.0	2.3	3.0	Berrebi et al. (1994)
Regeneration efficiency	Global	2009	70-80/>95	%	4.0	2.0	2.8	Rosso (2009)

Deactivation	Europe	2007	50	% (w)	4.0	2.3	3.0	Dufresne (2007)
Use loss	Global	2015	5	%	3.0	2.0	2.3	Ciacci et al. (2015)
Collection rate	USA	1980	50	% (w)	3.5	2.3	2.8	Shedd (1993)
Recycling efficiency	Europe	1994	97	% (w)	4.0	2.7	3.2	Berrebi et al. (1994)
Recycling efficiency	The Netherlands	Not available	>90	%	4.0	2.3	3.0	Marafi and Stanislaus (2008)

Table A6. Full dataset of the application hydroformylation and GTL process catalysts. It includes the DQA results in terms of R_L (reliability), R_P (representativeness) and DQR (data quality rating).

**Parameter**	**Data country/region**	**Data year**	**Value**	**Unit**	**DQA**			**Reference**
					**R_L**	**R_P**	**DQR**	
Processing yield	USA	1980	97	% (w)	3.5	2.7	3.0	Shedd (1993)
Use loss	Global	2015	9	%	3.0	2.0	2.3	Ciacci et al. (2015)
Collection rate	USA	1980	90	% (w)	2.5	2.3	2.4	National Research Council (1983)

Table A7. Full dataset of the application PET precursors catalysts. It includes the DQA results in terms of R_L (reliability), R_P (representativeness) and DQR (data quality rating).

**Parameter**	**Data country/region**	**Data year**	**Value**	**Unit**	**DQA**			**Reference**
					**R_L**	**R_P**	**DQR**	
Processing yield	USA	1980	97	% (w)	3.5	2.7	3.0	Shedd (1993)
Life time	USA	2005	8	years	4.0	2.3	3.0	Harper et al. (2011)
Life time	EU	2018	0.5	years	3.0	1.0	1.5	PET Manufacturers in Europe (2018)
Use loss	Global	2015	9	%	3.0	2.0	2.3	Ciacci et al. (2015)
Collection rate	EU	2018	50	% (w)	3.0	1.0	1.5	Committee of PET Manufacturers in Europe (2018)
Collection rate	USA	1980	50	% (w)	2.5	2.3	2.4	National Research Council (1983)
Recycling efficiency	USA	1998	80	%	3.0	2.7	2.8	Miserlis (2000)

Table A8. Full dataset of the application unspecified Co catalysts. It includes the DQA results in terms of R_L (reliability), R_P (representativeness) and DQR (data quality rating).

**Parameter**	**Data country/region**	**Data year**	**Value**	**Unit**	**DQA**			**Reference**
					**R_L**	**R_P**	**DQR**	
Processing yield	USA	1980	95.9	% (w)	2.5	2.7	2.6	National Research Council (1983)
Processing yield	USA	1980	97.1	% (w)	2.5	2.7	2.6	National Research Council (1983)
Manufacturing yield	USA	1980	95.7	% (w)	2.5	2.7	2.6	National Research Council (1983)
Manufacturing yield	USA	1980	97.0	% (w)	2.5	2.7	2.6	National Research Council (1983)
Production scrap recovery	USA	1980	0	% (w)	2.5	2.7	2.6	National Research Council (1983)
Production scrap recovery	EU	2012	100	% (w)	4.0	1.7	2.3	BIO by Deloitte (2015)
Life time	USA	1980	2	years	2.5	2.7	2.6	National Research Council (1983)
Hoarding rate	EU	2012	0	% (w)	4.0	1.7	2.3	BIO by Deloitte (2015)
Hoarding time	EU	2012	0	years	4.0	1.7	2.3	BIO by Deloitte (2015)
Collection rate	USA	2005	100	% (w)	4.0	2.0	2.5	BIO by Deloitte (2015)
Recycling efficiency	USA	1998	97	% (w)	3.5	3.3	3.4	Shedd (2004)
Recycling efficiency	EU	2012	85	% (w)	4.0	2.0	2.5	BIO by Deloitte (2015)

Table A9. Full dataset of the application dissipative uses. It includes the DQA results in terms of R_L (reliability), R_P (representativeness) and DQR (data quality rating).

**Parameter**	**Data country/region**	**Data year**	**Value**	**Unit**	**DQA**			**Reference**
					**R_L**	**R_P**	**DQR**	
Processing yield	USA	1980	97	% (w)	2.5	2.7	2.6	National Research Council (1983)

Manufacturing yield	USA	1980	95	% (w)	2.5	2.7	2.6	National Research Council (1983)
Manufacturing scrap recovery	Global	2005	100	% (w)	4.0	2.0	2.5	Harper et al. (2011)
Life time	Global	2005	1	years	4.0	2.3	2.8	Harper et al. (2011)
Life time	USA	1980	5	years	2.5	2.7	2.6	National Research Council (1983)
Life time	USA	1980	5-25	years	2.5	2.7	2.6	National Research Council (1983)
Life time	Japan	2012	5	years	4.0	2.0	2.5	Murao and Ono (2012)
Shape parameter (Weibull distribution)	Japan	2012	3.5	-	4.0	2.0	2.5	Murao and Ono (2012)
Hoarding rate	EU	2012	0	% (w)	4.0	1.7	2.3	BIO by Deloitte (2015)
Hoarding time	EU	2012	0	years	4.0	1.7	2.3	BIO by Deloitte (2015)
Non-selective collection rate	Global	2005	100	% (w)	4.0	2.0	2.5	Harper et al. (2011)
Collection rate	USA	2005	0	% (w)	4.0	2.3	2.8	Harper et al. (2011)

Table A10. Full dataset of the application hard metals. It includes the DQA results in terms of R_L (reliability), R_P (representativeness) and DQR (data quality rating).

**Parameter**	**Data country/region**	**Data year**	**Value**	**Unit**	**DQA** R_L	R_P	**DQR**	**Reference**
Processing yield	USA	1980	98.3	% (w)	2.5	2.3	2.4	National Research Council (1983)
Manufacturing yield	USA	1980	100	% (w)	2.5	2.3	2.4	National Research Council (1983)
Manufacturing yield	USA	1980	95	% (w)	3.5	2.3	2.8	Shedd (1993)
Manufacturing scrap recovery	USA	1980	100	% (w)	2.5	2.3	2.4	National Research Council (1983)
Life time	USA	1980	2	years	2.5	2.3	2.4	National Research Council (1983)
Life time	USA	2005	1	year	4.0	2.0	2.8	Harper et al. (2011)
Life time	Japan	2012	10.7	years	2.5	1.7	2.0	Nomura and Suga (2013)
Shape parameter (Weibull distribution)	Japan	2012	1.16	-	2.5	1.7	2.0	Nomura and Suga (2013)
Hoarding rate	Global	Not available	67	% (w)	4.0	2.0	2.8	BIO by Deloitte (2015)
Hoarding time	EU	2012	1.0	years	4.0	1.3	2.0	BIO by Deloitte (2015)
Non-selective collection rate	USA	2007	53	% (w)	3.5	2.0	2.6	BIO by Deloitte (2015)
Collection rate	USA	2007	47	% (w)	3.5	2.0	2.6	BIO by Deloitte (2015)
Collection rate	USA	1998	60	%	4.0	2.3	3.0	American Metal Market (2001)
Collection rate	Not available	Not available	50	%	4.0	1.7	2.6	Karhumaa and Kurkela (2013)
Collection rate	USA	2007	50	% (w)	3.5	2.0	2.6	Harper et al. (2011)
Collection rate	USA	2007	75	% (w)	3.5	2.0	2.6	Harper et al. (2011)
Collection rate	USA	2007	15	% (w)	3.5	2.0	2.6	Harper et al. (2011)
Collection rate	USA	1980	36	% (w)	3.5	2.3	2.8	Shedd (1993)
Pre-treatment efficiency	Korea	2017	77.3	% (w)	2.5	1.3	1.8	Lee et al. (2017)
Pre-treatment efficiency	EU	2018	Almost 100	% (w)	3.0	1.0	1.5	Wolfram (2018)
Distribution to recycling processes	USA	1996	80	%	4.0	2.3	3.0	Jana et al. (1996)
Distribution to recycling processes	Not available	Not available	58	%	3.5	2.3	2.8	Stjernberg and Johnson (1998)
	Not available	Not available	42	%	3.5	2.3	2.8	Stjernberg and Johnson (1998)
Distribution to recycling processes	Not available	Not available	67	%	4.0	2.3	3.0	Šándorová (2017)
	Not available	Not available	33	%	4.0	2.3	3.0	Šándorová (2017)
Recycling efficiency	EU	2012	85	% (w)	4.0	2.0	2.5	BIO by Deloitte (2015)
Recycling efficiency	EU	Not available	95	%	4.0	2.7	3.2	Kurylak et al. (2016)

Table A11. Full dataset of the application magnets. It includes the DQA results in terms of R_L (reliability), R_P (representativeness) and DQR (data quality rating).

Parameter	Data country/region	Data year	Value	Unit	DQA R_L	R_P	DQR	Reference
Processing yield	USA	1980	95.7	% (w)	2.5	2.3	2.4	National Research Council (1983)
Processing yield	USA	1980	89.9	% (w)	2.5	2.3	2.4	National Research Council (1983)
Processing yield	USA	1980	92.2	% (w)	2.5	2.3	2.4	National Research Council (1983)
Processing downcycled scrap	USA	1980	50	% (w)	2.5	2.3	2.4	National Research Council (1983)
Processing downcycled scrap	USA	1980	50	% (w)	2.5	2.3	2.4	National Research Council (1983)
Processing downcycled scrap	USA	1980	50	% (w)	2.5	2.3	2.4	National Research Council (1983)
Manufacturing yield	USA	1980	94.3	% (w)	2.5	2.3	2.4	National Research Council (1983)
Manufacturing yield	USA	1980	98.4	% (w)	2.5	2.3	2.4	National Research Council (1983)
Manufacturing yield	USA	1980	97.2	% (w)	2.5	2.3	2.4	National Research Council (1983)
Manufacturing yield	USA	2012	70-85	% (w)	4.0	1.7	2.6	Liu and Chinnasamy (2012)
Manufacturing yield	USA	2012	50	% (w)	4.0	1.7	2.6	Liu and Chinnasamy (2012)
Manufacturing yield	Not available	2013	70	% (w)	4.0	2.0	2.8	Binnemans et al. (2013)
Manufacturing scrap recovery	USA	1980	0	% (w)	2.5	2.3	2.4	National Research Council (1983)
Manufacturing scrap recovery	USA	1980	0	% (w)	2.5	2.3	2.4	National Research Council (1983)
Manufacturing scrap recovery	USA	1980	0	% (w)	2.5	2.3	2.4	National Research Council (1983)
Manufacturing downcycled scrap	USA	1980	0	% (w)	2.5	2.3	2.4	National Research Council (1983)
Manufacturing downcycled scrap	USA	1980	0	% (w)	2.5	2.3	2.4	National Research Council (1983)
Manufacturing downcycled scrap	USA	1980	0	% (w)	2.5	2.3	2.4	National Research Council (1983)
Life time	USA	1980	10	years	2.5	2.3	2.4	National Research Council (1983)
Life time	USA	2005	5	years	4.0	2.0	2.5	Harper et al. (2011)
Life time	Japan	2012	6.9	years	3.0	2.0	2.4	Nomura and Suga (2013)
Life time	Japan	2012	8.3	years	3.0	2.0	2.4	Nomura and Suga (2013)
Life time	Global	2016	6	years	4.0	2.0	2.5	Schulze and Buchert (2016)
Life time	Japan	2012	8	years	3.0	2.0	2.4	Nomura and Suga (2013)
Life time	Japan	2012	15.6	years	3.0	2.0	2.4	Nomura and Suga (2013)
Life time	Global	2016	22	years	4.0	2.0	2.5	Schulze and Buchert (2016)
Life time	Global	2014	20	years	4.0	2.0	2.5	Habib and Wenzel (2014)
Life time	Global	2016	20	years	4.0	2.0	2.5	Schulze and Buchert (2016)
Life time	Japan	2012	16.4	years	3.0	2.0	2.4	Nomura and Suga (2013)
Life time	Global	2016	15	years	4.0	2.0	2.5	Schulze and Buchert (2016)
Life time	Global	2016	13	years	4.0	2.7	3.0	Schulze and Buchert (2016)
Life time	Global	2014	10	years	4.0	2.0	2.5	Habib and Wenzel (2014)
Shape parameter (Weibull distribution)	Japan	2012	1.83	-	3.0	2.0	2.4	Nomura and Suga (2013)
Shape parameter (Weibull distribution)	Japan	2012	2.25	-	3.0	2.0	2.4	Nomura and Suga (2013)
Shape parameter (Weibull distribution)	Japan	2012	1.98	-	3.0	2.0	2.4	Nomura and Suga (2013)
Shape parameter (Weibull distribution)	Japan	2012	1.6	-	3.0	2.0	2.4	Nomura and Suga (2013)
Shape parameter (Weibull distribution)	Japan	2012	1.65	-	3.0	2.0	2.4	Nomura and Suga (2013)

Non-selective collection rate	EU 28 + Switzerland and Norway	2015	0	% (w)	2.5	2.0	2.2	Huisman et al. (2017)
Non-selective collection rate	EU 28 + Switzerland and Norway	2015	0.9	% (w)	2.5	2.0	2.2	Huisman et al. (2017)
Collection rate	USA	2005	10	% (w)	4.0	2.0	2.5	Harper et al. (2011)
Collection rate	Europe	2016	35	% (w)	2.5	2.0	2.2	Baldé et al. (2017)
Collection rate	EU 28 + Switzerland and Norway	2015	44.6	% (w)	2.5	2.0	2.2	Huisman et al. (2017)
Collection rate	EU 28 + Switzerland and Norway	2015	44.8	% (w)	2.5	2.0	2.2	Huisman et al. (2017)
Collection rate	Global	2016	90	% (w)	4.0	2.0	2.5	Schulze and Buchert (2016)
Collection rate	Global	2016	90	% (w)	4.0	2.0	2.5	Schulze and Buchert (2016)
Collection rate	Global	2016	60	% (w)	4.0	2.0	2.5	Schulze and Buchert (2016)
Collection rate	Global	2016	80	% (w)	4.0	2.0	2.5	Schulze and Buchert (2016)
Collection rate	Global	2016	80	% (w)	4.0	2.7	3.0	Schulze and Buchert (2016)
Pre-treatment efficiency	Global	2016	90	% (w)	4.0	2.0	2.5	Schulze and Buchert (2016)
Pre-treatment efficiency	Global	2016	90	% (w)	4.0	2.0	2.5	Schulze and Buchert (2016)
Pre-treatment efficiency	Global	2016	60	% (w)	4.0	2.0	2.5	Schulze and Buchert (2016)
Pre-treatment efficiency	Global	2016	90	% (w)	4.0	2.0	2.5	Schulze and Buchert (2016)
Pre-treatment efficiency	Global	2016	40	% (w)	4.0	2.7	3.0	Schulze and Buchert (2016)

Table A12. Full dataset of the application other metallic uses. It includes the DQA results in terms of R_L (reliability), R_P (representativeness) and DQR (data quality rating).

Parameter	Data country/region	Data year	Value	Unit	DQA<br>R_L	 R_P	 DQR	Reference
Processing yield	USA	1980	90	% (w)	2.5	2.7	2.6	National Research Council (1983)
Processing yield	USA	1973	78	% (w)	2.5	2.7	2.6	Curwick et al. (1980b)
Processing yield	USA	1976	78.7	% (w)	3.0	2.7	2.8	Curwick et al. (1980a)
Processing yield	USA	1980	97-99	% (w)	2.5	2.7	2.6	National Research Council (1983)
Processing yield	USA	1980	84	% (w)	2.5	2.7	2.6	National Research Council (1983)
Processing yield	USA	1976	50.1	% (w)	3.0	2.7	2.8	National Research Council (1983)
Processing scrap recovery	USA	1980	100	% (w)	2.5	2.7	2.6	National Research Council (1983)
Processing scrap recovery	USA	1973	36.4	% (w)	3.0	2.7	2.8	Curwick et al. (1980b)
Processing downcycled scrap	USA	1980	50	% (w)	2.5	2.7	2.6	National Research Council (1983)
Processing downcycled scrap	USA	1973	36.4	% (w)	3.0	2.7	2.8	Curwick et al. (1980b)
Manufacturing yield	USA	1980	63.6	% (w)	3.5	2.7	3.0	National Research Council (1983)
Manufacturing yield	USA	1973	68	% (w)	3.0	2.7	2.8	Curwick et al. (1980b)
Manufacturing yield	USA	1976	59.5	% (w)	3.0	2.7	2.8	Curwick et al. (1980a)
Manufacturing yield	USA	1980	97.6	% (w)	2.5	2.7	2.6	National Research Council (1983)
Manufacturing scrap recovery	USA	1980	47.6	% (w)	2.5	2.7	2.6	National Research Council (1983)
Manufacturing scrap recovery	USA	1973	90.6	% (w)	3.0	2.7	2.8	Curwick et al. (1980b)
Manufacturing downcycled scrap	USA	1980	47.6	% (w)	2.5	2.7	2.6	National Research Council (1983)
Manufacturing downcycled scrap	USA	1973	0	% (w)	3.0	2.7	2.8	Curwick et al. (1980b)
Life time	USA	1980	5	years	2.5	2.7	2.6	National Research Council (1983)
Life time	USA	Not availa ble	4.6 - 7.4	years	4.0	3.3	3.6	Ely (2014)

Life time	Japan	2002	13.4	years	3.0	3.3	3.2	Nomura (2005)
Life time	Japan	2002	7.3	years	3.0	2.7	2.8	Nomura (2005)
Life time	The Netherlands	2001	8.8	years	3.0	2.7	2.8	Van den Bergen et al. (2008)
Life time	Japan	2006	7.9	years	3.0	2.3	2.6	Nomura and Momose (2008)
Life time	Japan	2012	11.4	years	3.0	2.0	2.4	Nomura and Suga (2013)
Life time	Japan	2012	11.1	years	3.0	2.0	2.4	Nomura and Suga (2013)
Shape parameter (Weibull distribution)	The Netherlands	2001	1.77	-	3.0	2.7	2.8	Van den Bergen et al. (2008)
Shape parameter (Weibull distribution)	Japan	2002	1.74	-	3.0	3.3	3.2	Nomura (2005)
Shape parameter (Weibull distribution)	Japan	2002	2.46	-	3.0	2.7	2.8	Nomura (2005)
Shape parameter (Weibull distribution)	Japan	2006	1.39	-	3.0	2.3	2.6	Nomura and Momose (2008)
Shape parameter (Weibull distribution)	Japan	2012	1.37	-	3.0	2.0	2.4	Nomura and Suga (2013)
Shape parameter (Weibull distribution)	Japan	2012	1.57	-	3.0	2.0	2.4	Nomura and Suga (2013)
Non-selective collection rate	EU 28 + Switzerland and Norway	2015	6.9	% (w)	2.5	2.0	2.2	Huisman et al. (2017)
Collection rate	Europe	2016	35	% (w)	2.5	2.0	2.2	Baldé et al. (2017)
Collection rate	USA	1980	21	% (w)	2.5	2.7	2.6	National Research Council (1983)
Collection rate	UK	2001	15	% (w)	3.0	2.7	2.8	Goosey and Kellner (2002)
Collection rate	Global	2012	15	% (w)	4.0	3.0	3.4	Baldé et al. (2015)
Collection rate	EU27	2005	25-40	% (w)	3.0	2.7	2.8	Huisman et al. (2008)
Collection rate	EU	2014	35	%	4.0	2.0	2.8	Eurometaux (2016)

Table A13. Full dataset of the application superalloys. It includes the DQA results in terms of R_L (reliability), R_P (representativeness) and DQR (data quality rating).

**Parameter**	**Data country/region**	**Data year**	**Value**	**Unit**	**DQA**			**Reference**
					**R_L**	**R_P**	**DQR**	
Processing yield	USA	1976	80	% (w)	3.0	2.3	2.6	Curwick et al. (1980a)
Processing yield	USA	1976	50	% (w)	3.0	2.3	2.6	Curwick et al. (1980a)
Processing yield	USA	1976	54	% (w)	3.0	2.3	2.6	Curwick et al. (1980a)
Processing yield	USA	1976	79	% (w)	2.5	2.3	2.4	National Research Council (1983)
Processing yield	USA	1976	48	% (w)	2.5	2.3	2.4	National Research Council (1983)
Processing yield	USA	1980	94	% (w)	2.5	2.3	2.4	National Research Council (1983)
Processing yield	USA	1980	87	% (w)	2.5	2.3	2.4	National Research Council (1983)
Processing yield	USA	1980	91	% (w)	2.5	2.3	2.4	National Research Council (1983)
Processing yield	USA	1980	61	% (w)	3.0	2.3	2.6	Shedd (1993)
Processing yield	Global	2001	50-75	%	4.0	2.7	3.2	Donachie and Donachie (2002)
Processing scrap recovery	USA	1980	93	% (w)	3.0	2.3	2.6	Shedd (1993)
Processing scrap recovery	USA	1976	91	% (w)	3.0	2.3	2.6	Curwick et al. (1980a)
Processing scrap recovery	Global	2005	100	% (w)	4.0	2.0	2.5	Harper et al. (2011)
Processing downcycled scrap	USA	1980	69	% (w)	2.5	2.3	2.4	National Research Council (1983)
Processing downcycled scrap	USA	1980	50	% (w)	2.5	2.3	2.4	National Research Council (1983)
Processing downcycled scrap	USA	1980	57	% (w)	2.5	2.3	2.4	National Research Council (1983)
Processing downcycled scrap	USA	1976	5	% (w)	3.0	2.3	2.6	Curwick et al. (1980a)
Manufacturing yield	USA	1976	40	% (w)	3.0	2.3	2.6	Curwick et al. (1980a)
Manufacturing yield	USA	1976	54	% (w)	3.0	2.3	2.6	Curwick et al. (1980a)
Manufacturing yield	USA	1976	51	% (w)	3.0	2.3	2.6	Curwick et al. (1980a)

Manufacturing yield	USA	1980	40	% (w)	2.5	2.3	2.4	National Research Council (1983)
Manufacturing yield	USA	1980	53	% (w)	2.5	2.3	2.4	National Research Council (1983)
Manufacturing yield	USA	1980	48	% (w)	2.5	2.3	2.4	National Research Council (1983)
Manufacturing yield	USA	1980	65	% (w)	3.0	2.3	2.6	Shedd (1993)
Manufacturing scrap recovery	USA	1980	13	% (w)	2.5	2.3	2.4	National Research Council (1983)
Manufacturing scrap recovery	USA	1980	81	% (w)	2.5	2.3	2.4	National Research Council (1983)
Manufacturing scrap recovery	USA	1980	46	% (w)	2.5	2.3	2.4	National Research Council (1983)
Manufacturing scrap recovery	USA	1980	91	% (w)	3.0	2.3	2.6	Shedd (1993)
Manufacturing scrap recovery	USA	1976	30	% (w)	3.0	2.3	2.6	Curwick et al. (1980a)
Manufacturing downcycled scrap	USA	1980	81	% (w)	2.5	2.3	2.4	National Research Council (1983)
Manufacturing downcycled scrap	USA	1980	17	% (w)	2.5	2.3	2.4	National Research Council (1983)
Manufacturing downcycled scrap	USA	1980	50	% (w)	2.5	2.3	2.4	National Research Council (1983)
Manufacturing downcycled scrap	USA	1980	0	% (w)	3.0	2.3	2.6	Shedd (1993)
Manufacturing downcycled scrap	USA	1976	63	% (w)	3.0	2.3	2.6	Curwick et al. (1980a)
Life time	USA	1980	5	years	2.5	2.3	2.4	National Research Council (1983)
Life time	USA	2005	5	years	4.0	2.3	3.0	Harper et al. (2011)
Life time	Japan	2006	6	years	3.0	2.3	2.6	Nomura and Momose (2008)
Life time	Japan	2006	20	years	3.0	2.3	2.6	Nomura and Momose (2008)
Life time	Japan	2012	23	years	3.0	2.0	2.4	Nomura and Suga (2013)
Life time	Japan	2012	19	years	3.0	2.0	2.4	Nomura and Suga (2013)
Shape parameter (Weibull distribution)	Japan	2006	2.0	-	3.0	2.3	2.6	Nomura and Momose (2008)
Shape parameter (Weibull distribution)	Japan	2006	2.0	-	3.0	2.3	2.6	Nomura and Momose (2008)
Shape parameter (Weibull distribution)	Japan	2012	1.8	-	3.0	2.0	2.4	Nomura and Suga (2013)
Shape parameter (Weibull distribution)	Japan	2012	1.7	-	3.0	2.0	2.4	Nomura and Suga (2013)
Hoarding rate	USA	2010	100	% (w)	3.5	2.0	2.6	Jiang (2013)
Hoarding time	USA	2010	5	years	3.5	2.0	2.6	Jiang (2013)
Non-selective collection rate	USA	1980	21	% (w)	2.5	2.3	2.4	National Research Council (1983)
Non-selective collection rate	USA	1980	17	% (w)	2.5	2.3	2.4	National Research Council (1983)
Non-selective collection rate	USA	1980	15	% (w)	3.0	2.3	2.6	Shedd (1993)
Collection rates	EU	2012	100	% (w)	4	1.7	2.3	BIO by Deloitte (2015)
Collection rate	USA	2012	90	%	4.0	1.7	2.6	Reck and Graedel (2012)
Collection rate	USA	1973	50-60	% (w)	3.0	2.3	2.6	Curwick et al. (1980b)
Collection rate	USA	1980	50	% (w)	2.5	2.3	2.4	National Research Council (1983)
Collection rate	USA	1980	50	% (w)	2.5	2.3	2.4	National Research Council (1983)
Collection rate	USA	1980	50	% (w)	3.0	2.3	2.6	Shedd (1993)
Collection rate	USA	2010	90	% (w)	4.0	2.3	3.0	Harper et al. (2011)
Distribution to recycling processes	USA	2012	80/20	%	4.0	1.7	2.6	Reck and Graedel (2012)
Distribution to recycling processes	USA	1980	63/37	% (w)	2.5	2.3	2.4	National Research Council (1983)
Distribution to recycling processes	USA	1980	60/40	% (w)	3.5	2.3	2.8	National Research Council (1983)
Distribution to recycling processes	USA	1980	59/41	% (w)	1.5	2.3	2.0	Shedd (1993)
Recycling efficiency	USA	2012	76	% (w)	4.0	2.0	2.8	BIO by Deloitte (2015)

5. List of consulted experts

Fifteen experts from academia; 18 associations, groups or societies; and more than 110 companies (producers, users, and recyclers) were contacted for collection and/or validation of data. Of these, 51 gave a general answer. Table A14 lists the institutions that provided an expert insight about Co flows, and/or information used in data collection or validation. Some of these institutions and/or experts asked not to reveal their identity.

Table A14. List of companies, associations, and experts consulted for data collection and/or validation.

Material or application	Affiliation	Expert	Position	Information about Co flows	Information used in data collection or validation
Batteries	Accurec	Anonymous	-	No	No
	Undisclosed company	Anonymous	-	Yes	Yes
	EBRA (European Battery Recycling Association)	Alain Vassart	Secretary General of EBRA ivzw/aisbl	Yes	Yes
	EPBA (European Portable Battery Association)	Anonymous	-	Yes	No
	EUROBAT (Association of European Automotive and Industrial Battery Manufacturers)	Anonymous	-	No	No
	FFE (Forschungsstelle für Energiewirtschaft)	Anonymous	-	No	No
	JRC (Joint Research Centre)	Silvia Bobba	PhD researcher	Yes	No
	Saubermacher AG	Anonymous	-	Yes	Yes
Catalysts	Clariant	Thomas Cotter	BU Catalysts, Emission Control and Zeolites R&D Department Manager	Yes	No
	CPME (Committee of PET Manufacturers in Europe)	Anonymous	-	Yes	Yes
	European Catalyst Manufacturers Association	Anonymous	-	No	No
Cobalt and other materials	BRGM (Bureau de Recherches Géologiques et Minières)	Raphael Danino-Perraud	PhD researcher	Yes	Yes
	CI (Cobalt Institute)	Carol-lynne Pettit	REACH & Sustainability Manager	Yes	Yes
		David Weight	President	Yes	Yes
	CMI (Critical Material Institute)	Roderick Eggert	Deputy Director	Yes	Yes
	DERA (German Mineral Resources Agency)	Siyamend Ingo Al Barazi	Scientist	Yes	Yes
	Empa	Anonymous	-	No	No
	Ghent University	Stijn Dewaele	Professor	Yes	No
	Glencore – Nikkelverk	Oluf Bøckman	Senior Specialist, R&D	Yes	Yes
	IMA-Europe (Industrial Minerals)	Anonymous	-	No	No
	JRC (Joint Research Centre)	Anonymous	-	No	No

Cobalt and other materials	KIT (Karlsruhe Institute of Technology)	Anonymous	-	No	No
		Anonymous	-	No	Yes
	KU Leuven	Anonymous	-	No	No
	Leiden University	Sebastiaan Deetman	PhD researcher	Yes	No
	Kanva EU Ltd	Andrejs Kopils	Development Director	Yes	Yes
		Sergejs Kopils	Managing Director	Yes	Yes
	Undisclosed company	Anonymous	-	No	No
		Anonymous	-	No	No
	NTNU (Norwegian University of Science and Technology)	Anonymous	-	Yes	No
	PNO Consultants	Anonymous	-	No	No
	Umicore	Anonymous	-	Yes	No
		Wouter Ghyoot	Director Sustainable Value Chain	Yes	No
	Yale University	Anonymous	-	No	No
Dissipative uses	Inorganic Pigments Consortium	Anonymous	-	No	No
Hard metals	Undisclosed company	Anonymous	-	Yes	Yes
	EuroHM (European Hard Materials Group)	Steven Moseley	Chief Scientist – Hard Materials	No	No
	Oerlikon	Anonymous	-	No	No
	Sumitomo	Anonymous	-	No	No
	Wolfram	Michael Dornhofer	Director Sales & Purchasing	Yes	Yes
Magnets	Goudsmit Magnetics	Anonymous	-	No	No
	UK Magnetics Society	Anonymous	-	Yes	Yes
Other metallic uses	ACB	Eddy Geerinckx	-	Yes	No
	Dutch Surface Treatment Association	Anonymous	-	No	No
	Undisclosed association	Anonymous	-	No	No
	Euro circuits	Anonymous	-	Yes	No
	German Surface Treatment Association	Anonymous	-	No	No
	Multi-CB	Anonymous	-	No	No
	Schmolz + Bickenbach Group	Anonymous	-	No	No
	Surface Engineering Association	Anonymous	-	No	No
Superalloys	Doncasters	Anonymous	-	No	No
Other	Undisclosed company	Anonymous	-	No	No
	Composite Recycling	Frank Riedewald	CEO	No	No
	RELIGHT	Anonymous	-	Yes	No
	Renewi	Anonymous	-	No	No
	Stena	Anonymous	-	Yes	No
	The Shift	Anonymous	-	No	No

Dataset references

Ahmadi, L., Young, S. B., Fowler, M., & Fraser, R. A. (2017). A cascaded life cycle: reuse of electric vehicle lithium-ion battery packs in energy storage systems. The International Journal of Life Cycle Assessment, 111–124. https://doi.org/10.1007/s11367-015-0959-7.

American Metal Market (2001). Several factors seen transforming scrap industry. American Metal Market Metals Recycling Supplement, 109(56), p. 10A.

Asari, M., & Sakai, S. (2013). Li-ion battery recycling and cobalt flow analysis in Japan. Resources, Conservation and Recycling, 81, 52–59. https://doi.org/10.1016/j.resconrec.2013.09.011.

Baldé, C.P., Wang, F., Kuehr, R., Huisman, J. (2015). The global e-waste monitor – 2014, United Nations University, IAS – SCYCLE, Bonn, Germany, 41p.

Baldé, C.P., Forti V., Gray, V., Kuehr, R., Stegmann, P. (2017). The Global E-waste Monitor – 2017, United Nations University (UNU), International Telecommunication Union (ITU) & International Solid Waste Association (ISWA), Bonn/Geneva/Vienna, 116 p.

Berrebi, G., Dufresne, P., & Jacquier, Y. (1994). Recycling of spent hydroprocessing catalysts: EURECAT technology. Resources, Conservation and Recycling, 10(1), 1–9. https://doi.org/10.1016/0921-3449(94)90032-9.

Binnemans, K., Jones, P. T., Blanpain, B., Van Gerven, T., Yang, Y., Walton, A., & Buchert, M. (2013). Recycling of rare earths: a critical review. Journal of Cleaner Production, 51, 1–22. https://doi.org/10.1016/j.jclepro.2012.12.037.

BIO by Deloitte (2015). Study on Data for a Raw Material System Analysis: Roadmap and Test of the Fully Operational MSA for Raw Materials. Prepared for the European Commission, DG GROW, 179 p.

Buchert, M., Manhart, A., Bleher, D., & Pingel, D. (2012). Recycling critical raw materials from waste electronic equipment. Commissioned by the North Rhine- Westphalia State Agency for Nature, Environment and Consumer Protection, 88 p.

Ciacci, L., Reck, B. K., Nassar, N. T., & Graedel, T. E. (2015). Lost by Design. Environmental Science & Technology, 49(16), 9443–9451. https://doi.org/10.1021/es505515z.

Circular Energy Storage (2018). The lithium-ion battery end-of-life market 2018-2025. UK, 128 p.

Committee of PET Manufacturers in Europe (2018). Personal communication (August 2018).

Contestabile, M., Panero, S., & Scrosati, B. (2001). A laboratory-scale lithium-ion battery recycling process. Journal of Power Sources, 92(1), 65–69. https://doi.org/10.1016/S0378-7753(00)00523-1.

Curwick, L.R., Petersen, W.A., & deBarbadillo, J.J. (1980a). Superalloy scrap-generation and recycling. Proceedings Superalloys 1980 (Fourth International Symposium), 21-30.

Curwick, L.R., Petersen, W.A., & Makar, H.V. (1980b). Availability of Critical Scrap metals Containing Chromium in the United States, Superalloys and Cast Heat and Corrosion Resistant Alloys. Information Circular 8821, U.S. Bureau of Mines, 51 p.

Dai, Q., Dunn, J., Kelly, J. C., & Elgowainy, A. (2017). Update of Life Cycle Analysis of Lithium-ion Batteries in the GREET Model. Argonne National Laboratory, USA, 18 p.

Darton Commodities Limited (2018). Cobalt Market Review. UK, 59 p.

Desmet, B. & Colin, J. (2017). How battery life cycle influences the collection rate of battery collection schemes. EUCOBAT – Mobius, 40 p.

Dewulf, J., Van der Vorst, G., Denturck, K., Van Langenhove, H., Ghyoot, W., Tytgat, J., & Vandeputte, K. (2010). Recycling rechargeable lithium ion batteries: Critical analysis of natural resource savings.

Resources, Conservation and Recycling, 54(4), 229–234. https://doi.org/10.1016/j.resconrec.2009.08.004.

Donachi, M.J. & Donachi, S.J (2002). SUPERALLOYS A Technical Guide, 2nd ed. USA: ASM International, 402 p.

Dufresne, P. (2007). Hydroprocessing catalysts regeneration and recycling. Applied Catalysis A: General, 322, 67–75. https://doi.org/10.1016/j.apcata.2007.01.013.

EBRA (2018). Personal communication (July 2018).

Ely, C. (2014). The Life Expectancy of Electronics [online] <https://www.cta.tech/News/Blog/Articles/2014/September/The-Life-Expectancy-of-Electronics.aspx> Last view: 18/04/2019.

Environmental Protection Agency (2013). Application of Life-Cycle Assessment to Nanoscale Technology: Lithium-ion Batteries for Electric Vehicles. USA, 126 p.

EUROBAT (2014). A review of battery technologies for automotive applications. 72 p.

Eurometaux (2016). EU Circular Economy Package: Action Plan.

Georgi-Maschler, T., Friedrich, B., Weyhe, R., Heegn, H., & Rutz, M. (2012). Development of a recycling process for Li-ion batteries. Journal of Power Sources, 207, 173–182. https://doi.org/10.1016/j.jpowsour.2012.01.152

Goosey, M., & Kellner, R. (2002). A Scoping Study End-of-Life Printed Circuit Boards.

Habib, K., & Wenzel, H. (2014). Exploring rare earths supply constraints for the emerging clean energy technologies and the role of recycling. Journal of Cleaner Production, 84, 348–359. https://doi.org/10.1016/j.jclepro.2014.04.035.

Harper, E. M., Kavlak, G., & Graedel, T. E. (2012). Tracking the Metal of the Goblins: Cobalt's Cycle of Use. Environmental Science & Technology, 46(2), 1079–1086. https://doi.org/10.1021/es201874e.

Heegn, H., Friedrich, B., Müller, T., & Weyhe, R. (2003). Closed-Loop Recycling of Nickel, Cobalt and Rare Earth Metals from spent Nickel-Metal Hydride-Batteries. XXII International Mineral Processing Congress, paper 36 OP39B.

Huisman, J., Magalini, F., Kuehr, R., Maurer, C., Ogilvie, S., Poll, J., Delgado, C., Artim, E., Szlezak, J., & Stevels, A. (2008). Review of Directive 2002/96 on Waste Electrical and Electronic Equipment (WEEE). Final Report. United Nations University, Bonn, Germany, 377p.

Huisman, J., Leroy, P., Tertre, F., Söderman, M.L., Chancerel, P., Cassard, D., Løvik, A,N., Wäger, P., Kushnir, D., Rotter, V.S., Mählitz, P., Herreras, L., Emmerich, J., Hallberg, A., Habib, H., Wagner, M., & Downes, S. (2017). Prospecting Secondary Raw Materials in the Urban Mine and mining wastes (ProSUM) - Final Report, ISBN: 978-92-808-9060-0 (print), 978-92-808-9061-7 (electronic), Belgium.

IVF Industrial Research and Development Corporation (2007). Lot 3 Personal Computers (desktops and laptops) and Computer Monitors. Prepared for the European Commission, DG TREN. Final report, 325 p.

Jana, R.K., Kumar, V., Saha, A.K., Rao, K.V., Pandey, B.D., & Premchand (1996). Processing of Tungsten Alloy Scrap for the Recovery of Tungsten Metal. In: Proceedings of National Seminar on Environmental & Waste Management in Metallurgical Industries, NS-EWM 1996, 94–98.

Jiang, H. (2013) Key findings on Airplane economic life. Boeing, 9 p.

Jiao, N., & Evans, S. (2016). Business Models for Sustainability: The Case of Second-life Electric Vehicle Batteries. Procedia CIRP, 40, 250–255. https://doi.org/10.1016/j.procir.2016.01.114.

Kantar WorldPanel (2016). Double Digit Smartphone Market Growth is Over [online] <https://www.kantarworldpanel.com/global/News/Double-Digit-Smartphone-Market-Growth-is-over> Last view: 17/04/2019.

Karhumaa, T., & Kurkela, M. (2013). Review of the hard metal recycling market and the role of the zinc process as a recycling option. Proceedings of the 18th Plansee Seminar, Reutte, Austria. Kneringer, G., Rodhammer, P., and Wildner, H. (eds). pp. 13/1–13/11.

Knights, B.D.H., & Saloojee, F. (2015). Lithium Battery Recycling – keeping the future fully charged. Green Economy Research Report No. 1, Green Fund, Development Bank of Southern Africa, Midrand.

Kumar, N., & Suman, Y. (2017). Cobalt recovery from waste li-ion batteries: development and issues in technology transfer. Indian J.Sci.Res. 7(2), 201–208.

Kurylak, W., Retegan, T., Bru, K., Mennade, N., Cassayre, L., Sundqvist, L., Ye, G., Yang, J., Koffeman, J., Yang, Y., Leszczynska-Sejda, K., & Benke, G. (2016). State of the art on the recovery of refractory metals from urban mines. MSP-Refram, 30 p.

Kushnir, D. (2015) Lithium Ion Battery Recycling Technology 2015: Current State and Future Prospects. Environmental Systems Analysis. Chalmers University, Göteborg, Sweden. ESA REPORT # 2015:18.

Lebedeva, N., Di Persio, F., Boon-Brett, L. (2016). Lithium ion battery value chain and related opportunities for Europe, European Commission, Petten, 80 p.

Lee, J., Kim, S., & Kim, B. (2017). A New Recycling Process for Tungsten Carbide Soft Scrap That Employs a Mechanochemical Reaction with Sodium Hydroxide. Metals, 7(7), 230. https://doi.org/10.3390/met7070230.

Liu, J., & Chinnasamy, C. (2012). Rare Earth Magnet Recycling. Rare Earth Elements Workshop, Colorado, USA.

Marafi, M., & Stanislaus, A. (2008). Spent hydroprocessing catalyst management: A review: Part II. Advances in metal recovery and safe disposal methods. Resources, Conservation and Recycling, 53(1), 1–26. https://doi.org/10.1016/j.resconrec.2008.08.005.

Mathieux, F., Ardente, F., Bobba, S., Nuss, P., Blengini, G., Alves Dias, P., Blagoeva, D., Torres De Matos, C., Wittmer, D., Pavel, C., Hamor, T., Saveyn, H., Gawlik, B., Orveillon, G., Huygens, D., Garbarino, E., Tzimas, E., Bouraoui, F., & Solar, S. (2017). Critical Raw Materials and the Circular Economy – Background report. JRC Science-for-policy report, EUR 28832 EN, Publications Office of the European Union, Luxembourg, ISBN 978-92-79-74282-8, doi:10.2760/378123 JRC108710.

Meskers, C., & Hagelüken, C. (2009). Green recycling of EEE: Special and precious metal recovery from EEE. Proceedings EPD Congress 2009, USA.

Miserlis, C. (2000). United States Patent No. 6117207. United States Patent Office.

Müller, T., & Friedrich, B. (2006). Development of a recycling process for nickel-metal hydride batteries. Journal of Power Sources, 158(2), 1498–1509. https://doi.org/10.1016/j.jpowsour.2005.10.046.

Murao, S., & Ono, K. (2012). Current status and future of lead-based paints and pigments in Asia and the Pacific. Interim report, Japan, 22p.

National Research Council (1983). Cobalt Conservation through Technological Alternatives. Washington D.C., National Academy Press, 205 p.

Nomura, K. (2005). Duration of Assets: Examination of Directly Observed Discard Data in Japan. KEO Discussion Paper No.99, 36 p.

Nomura, K., & Momose, F. (2008). Measurement of Depreciation Rates based on Disposal Asset Data in Japan. OECD Working Party on National Accounts, 25 p.

Nomura, K., & Suga, Y. (2013). Asset Service Lives and Depreciation Rates based on Disposal Data in Japan. Economic Measurement Group Workshop Asia, 29 p.

Oguchi, M., Kameya, T., Tasaki, T., Tamai, N., & Tanikawa, N. (2006). Estimation of Lifetime Distributions and Waste Numbers of 23 Types of Electrical and Electronic Equipment. Journal of the Japan Society of Waste Management Experts, 17(1), 50–60. https://doi.org/10.3985/jswme.17.50.

Reck, B.K., & Graedel, T.E. (2012). Challenges in Metal Recycling. Science, 337(6095), 690 LP-695. https://doi.org/10.1126/science.1217501.

Rosso, I.P. (2009). TRICAT Triage: Innovative Management of Spent HDS Catalyst, Recycling Metals from Industrial Waste, Colorado School of Mines, Golden, USA.

Saeki, S., Lee, J., Zhang, Q., & Saito, F. (2004). Co-grinding $LiCoO_2$ with PVC and water leaching of metal chlorides formed in ground product. International Journal of Mineral Processing, 74, S373–S378. https://doi.org/10.1016/j.minpro.2004.08.002.

Šándorová, K. (2017). Recycling of waste containing tungsten and its recovery. Research Journal of Mining, 1(3), 129–138.

Saubermacher Dienstleistungs AG (2018). Personal communication (July 2018).

Schulze, R., & Buchert, M. (2016). Estimates of global REE recycling potentials from NdFeB magnet material. Resources, Conservation and Recycling, 113, 12–27. https://doi.org/10.1016/j.resconrec.2016.05.004.

Shedd, K.B. (1993). The Materials Flow of Cobalt in the United States. U.S. Bureau of Mines, Information circular 9350, 31 p.

Shedd, K.B. (2004). Cobalt Recycling in the United States in 1998. In Sibley, S.F. (edit.) Flow studies for recycling metal commodities in the United States. U.S. Department of the Interior, Circular 1196–A–M.

Sommer, P., Rotter, V. S., & Ueberschaar, M. (2015). Battery related cobalt and REE flows in WEEE treatment. Waste Management, 45, 298–305. https://doi.org/10.1016/j.wasman.2015.05.009.

Stjernberg, Klas, & Johnson, John, Jr. (1998). Recycling of cemented carbides, 1998 International Conference on Powder Metallurgy & Particulate Materials. Proceedings: Princeton, N.J., Metal Powder Industries Federation and APMI International, pt. 1, p. 173–179.

Swart, P., Dewulf, J., & Biernaux, A. (2014). Resource demand for the production of different cathode materials for lithium ion batteries. Journal of Cleaner Production, 84, 391–399. https://doi.org/10.1016/j.jclepro.2014.01.056.

thinkstep AG (2017). PEFCR - Product Environmental Footprint Category Rules on High Specific Energy Rechargeable Batteries for Mobile Applications. Prepared for the European Commission, DG Environment, 104 p.

Tran, H. P., Schaubroeck, T., Swart, P., Six, L., Coonen, P., & Dewulf, J. (2018). Recycling portable alkaline/ZnC batteries for a circular economy: An assessment of natural resource consumption from a life cycle and criticality perspective. Resources, Conservation and Recycling, 135, 265–278. https://doi.org/10.1016/j.resconrec.2017.08.018.

Tytgat, J., & Van Damme, G. (2018). Towards an EU Battery Industry: How can recycling contribute to Europe’s ambitions?. Abstract book, Circular Economy of Battery Production and Recycling Conference 2018, Gothenburg, Sweden.

Van den Bergen, D., de Haan, M., de Heij, R., & Horsten, M. (2008). Measuring Capital in the Netherlands, 2005 OECD Working Party on National Accounts.

Weyhe, R. (2008). Final Report of the Research Project Recovery of Raw Materials from Li-ion Accumulators, ACCUREC Recycling GmbH. Supported by the German Federal Ministry of Education and Research, Support Code 01RW0405, Mülheim a. d. R.

Wilburn, D.R. (2008). Material use in the United States—Selected case studies for cadmium, cobalt, lithium, and nickel in rechargeable batteries. U.S. Geological Survey Scientific Investigations Report 2008–5141, 19 p.

Wolfram (2018). Personal communication (May 2018).

Ziemann, S., Müller, D.B., Schebek, L., & Weil, M. (2018). Modeling the potential impact of lithium recycling from EV batteries on lithium demand: A dynamic MFA approach. Resources, Conservation and Recycling, 133, 76–85. https://doi.org/10.1016/j.resconrec.2018.01.031.

References

A&A Coatings (2018). What Is Hardfacing? [online] <https://www.thermalspray.com/what-is-hardfacing/> Last view: 03/04/2019.

Al-Thyabat, S., Nakamura, T., Shibata, E., & Iizuka, A. (2013). Adaptation of minerals processing operations for lithium-ion (LiBs) and nickel metal hydride (NiMH) batteries recycling: Critical review. Minerals Engineering, 45, 4–17. https://doi.org/10.1016/j.mineng.2012.12.005.

Benecki, W. (2017). More Than You Ever Wanted to Know About the Permanent Magnet Industry!. The International Forum on Magnetic Applications, Technologies & Materials, Orlando, USA.

Brumby, A., Verhelst, M., & Cheret, D. (2005). Recycling GTL catalysts—A new challenge. Catalysis Today, 106(1), 166–169. https://doi.org/10.1016/j.cattod.2005.07.177.

Cobalt Institute (2018). Core Applications [online] <https://www.cobaltinstitute.org/core-applications.html> Last view: 03/04/2019.

Darton Commodities Limited (2018). Cobalt Market Review. UK, 59 p.

De, C., Saha, R., Ghosh, S. K., Ghosh, A., Mukherjee, K., Bhattacharyya, S. S., & Saha, B. (2013). A review of biphasic hydroformylation for long chain substrates. Research on Chemical Intermediates, 39(8), 3463–3474. https://doi.org/10.1007/s11164-012-0891-4.

Donachi, M.J. & Donachi, S.J (2002). SUPERALLOYS A Technical Guide, 2nd ed. USA: ASM International, 402 p.

Donaldson, J. D., & Beyersmann, D. (2012). Cobalt and Cobalt Compounds. In Ullmann's Encyclopedia of Industrial Chemistry, (Ed.). DOI: 10.1002/14356007.a07_281.pub2.

Dry, M. E. (1999). Fischer – Tropsch reactions and the environment. Applied Catalysis A: General 189(2), 185–190. https://doi.org/10.1016/S0926-860X(99)00275-6.

Dufresne, P. (2007). Hydroprocessing catalysts regeneration and recycling. Applied Catalysis A: General, 322, 67–75. https://doi.org/10.1016/j.apcata.2007.01.013.

Edelen, A., & Ingwersen, W. (2016). Guidance on Data Quality Assessment for Life Cycle Inventory Data. U.S. Environmental Protection Agency.

European Commission (2006). Directive 2006/66/EC of the European Parliament and of the Council on batteries and accumulators and waste batteries and accumulators and repealing Directive 91/157/EEC.

European Environment Agency (2016). Electric vehicles in Europe. Denmark, 74 p. doi: 10.2800/100230.

European Parliament (2006). DIRECTIVE 2012/19/EU on waste electrical and electronic equipment (WEEE).

Freemantle, C. S., Sacks, N., Topic, M., & Pineda-Vargas, C. A. (2014). Impurity characterization of zinc-recycled WC-6 wt.% Co cemented carbides. International Journal of Refractory Metals and Hard Materials, 44, 94–102. https://doi.org/https://doi.org/10.1016/j.ijrmhm.2014.01.019.

Hebrard, F., & Kalck, P. (2009). Cobalt-Catalyzed Hydroformylation of Alkenes: Generation and Recycling of the Carbonyl Species, and Catalytic Cycle. Chemical Reviews, 109(9), 4272–4282. https://doi.org/10.1021/cr8002533.

Hoornweg D., & Bhada-Tata, P. (2012). WHAT A WASTE A Global Review of Solid Waste Management. Urban Development and Local Government Unit of the Sustainable Development Network, World Bank.

International Molybdenum Association (2018). Tool & High Speed Steel [online] <https://www.imoa.info/molybdenum-uses/molybdenum-grade-alloy-steels-irons/tool-high-speed-steel.php> Last view: 03/04/2019.

Jahangiri, H., Bennett, J., Mahjoubi, P., Wilson, K., & Gu, S. (2014). A review of advanced catalyst development for Fischer–Tropsch synthesis of hydrocarbons from biomass derived syn-gas. Catalysis Science & Technology, 4(8), 2210–2229. https://doi.org/10.1039/C4CY00327F.

Joo, S.-H., Shin, D. J., Oh, C. H., Wang, J.-P., & Shin, S. M. (2016). Re-manufacture of cobalt-manganese-bromide as a liquid catalyst from spent catalyst containing cobalt generated from petrochemical processes via hydrometallurgy. Journal of Hazardous Materials, 318, 24–31. https://doi.org/https://doi.org/10.1016/j.jhazmat.2016.06.008.

Kurylak, W., Retegan, T., Bru, K., Mennade, N., Cassayre, L., Sundqvist, L., Ye, G., Yang, J., Koffeman, J., Yang, Y., Leszczynska-Sejda, K., Benke, G. (2016). State of the art on the recovery of refractory metals from urban mines. MSP-REFRAM.

Lai, C.-D., Murthy, D. N., & Xie, M. (2006). Weibull Distributions and Their Applications. In H. Pham (Ed.), Springer Handbook of Engineering Statistics (pp. 63–78). London: Springer London. https://doi.org/10.1007/978-1-84628-288-1_3.

Laner, D., Feketitsch, J., Rechberger, H., & Fellner, J. (2015). A Novel Approach to Characterize Data Uncertainty in Material Flow Analysis and its Application to Plastics Flows in Austria. Journal of Industrial Ecology, 00(0), 1-14. https://doi.org/10.1111/jiec.12326.

Manfredi, S., Allacker, K., Chomkhamsri, K., Pelletier, N., & Maia de Souza, D. (2012). Product Environmental Footprint (PEF) Guide. Prepared for the European Commission, DG Environment.

National Institute for Environmental Studies (2018). Lifespan definition [online] <http://www.nies.go.jp/lifespan/e_01.html> Last view: 03/04/2019.

National Research Council (1983). Cobalt Conservation through Technological Alternatives. Washington D.C., National Academy Press, 205 p.

Reck, B. K., & Graedel, T. E. (2012). Challenges in Metal Recycling. Science, 337(6095), 690 LP-695. https://doi.org/10.1126/science.1217501.

Shedd, K.B. (2004). Cobalt Recycling in the United States in 1998. In Sibley, S.F. (edit.) Flow studies for recycling metal commodities in the United States. U.S. Department of the Interior, Circular 1196–A–M.

Sinha, M. K., Pramanik, S., Kumari, A., Sahu, S.K., Prasad, L.B., Jha, M.K., Yoo, K., & Pandey, B.D. (2017). Recovery of value added products of Sm and Co from waste SmCo magnet by hydrometallurgical route. Separation and Purification Technology, 179, 1–12. https://doi.org/10.1016/j.seppur.2017.01.056.

Sommer, P., Rotter, V. S., & Ueberschaar, M. (2015). Battery related cobalt and REE flows in WEEE treatment. Waste Management, 45, 298–305. https://doi.org/10.1016/j.wasman.2015.05.009.

Srivastava, R. R., Kim, M., Lee, J., Jha, M. K., & Kim, B.-S. (2014). Resource recycling of superalloys and hydrometallurgical challenges. Journal of Materials Science, 49(14), 4671–4686. https://doi.org/10.1007/s10853-014-8219-y.

UNEP (2011). Recycling Rates of Metals. ISBN: 978-92-807-3161-3.

Weidema, B.P., & Wesnaes, M.S. (1996). Data quality management for life cycle inventories-an example of using data quality indicators. Journal of Cleaner Production 4(3), 167–174.

Wilson, G. T., Smalley, G., Suckling, J. R., Lilley, D., Lee, J., & Mawle, R. (2017). The hibernating mobile phone: Dead storage as a barrier to efficient electronic waste recovery. Waste Management, 60, 521–533. https://doi.org/https://doi.org/10.1016/j.wasman.2016.12.023.
